# Introducing a Bayesian model of selective attention based on active inference

**DOI:** 10.1038/s41598-019-50138-8

**Published:** 2019-09-26

**Authors:** M. Berk Mirza, Rick A. Adams, Karl Friston, Thomas Parr

**Affiliations:** 1 Wellcome Centre for Human Neuroimaging, Institute of Neurology, University College London, London, UK; 2 Department of Neuroimaging, Institute of Psychiatry, Psychology and Neuroscience, King’s College London, London, UK; 3 The NIHR Maudsley Biomedical Research Centre (BRC) at South London and Maudsley NHS Foundation Trust and the Institute of Psychiatry, Psychology and Neuroscience, King’s College London, London, UK; 4 Institute of Cognitive Neuroscience, 17 Queen Square, University College London, London, UK; 5 Division of Psychiatry, 149 Tottenham Court Road, University College London, London, UK; 6 Max Planck-UCL Centre for Computational Psychiatry and Ageing Research, 10-12 Russell Square, London, WC1B 5EH UK; 7 Department of Computer Science, University College London, Malet Place, London WC1E 7JE UK

**Keywords:** Computational neuroscience, Information theory and computation

## Abstract

Information gathering comprises actions whose (sensory) consequences resolve uncertainty (i.e., are salient). In other words, actions that solicit salient information cause the greatest shift in beliefs (i.e., information gain) about the causes of our sensations. However, not all information is relevant to the task at hand: this is especially the case in complex, naturalistic scenes. This paper introduces a formal model of *selective attention* based on active inference and *contextual* epistemic foraging. We consider a visual search task with a special emphasis on goal-directed and task-relevant exploration. In this scheme, attention modulates the expected fidelity (precision) of the mapping between observations and hidden states in a state-dependent or context-sensitive manner. This ensures task-irrelevant observations have little expected information gain, and so the agent – driven to reduce expected surprise (i.e., uncertainty) – does not actively seek them out. Instead, it selectively samples task-relevant observations, which inform (task-relevant) hidden states. We further show, through simulations, that the atypical exploratory behaviours in conditions such as autism and anxiety may be due to a failure to appropriately modulate sensory precision in a context-specific way.

## Introduction

Our aim is to provide a ‘first principle’ account of endogenous attention that emphasises the intimate relationships between overt attention and saccadic searches, and covert attention and salience. Imagine that you are at your desk and looking around for your red pen. How could one account for the ensuing saccadic eye movements from first principles. The basic idea pursued below is that every action – overt or covert – is in the game of resolving uncertainty by sampling the sensorium to reduce expected surprise (or a variational bound on surprise called free energy). This is known as active inference and requires us to commit to actions that have the greatest epistemic affordance or information gain. So how does this explain how we look for a ‘red pen’?

Put simply, if we are compelled to sample the most salient, uncertainty reducing part of the visual scene, then it is sufficient to increase the precision or informativeness of the visual features that a ‘red pen’ possesses – and decrease the precision of all other features. More colloquially, it is sufficient to render ‘redness’ and ‘pen-ness’ more salient. Notice that there is a fundamental distinction between making some attributes more salient – by biasing their contribution to epistemic affordance – and simply adjusting the attentional gain (i.e., precision) of different sensory streams. In this model of selective (i.e., endogenous) attention, the selection is proactive: it is mediated by making certain sensations more attractive to sample, before they are actually sampled. In this sense, selective attention becomes part of planning a saccadic eye movement, not simply attending to the sensory consequences of that movement. This reading of selective attention is closely related to premotor theories of attention^1^ – that emphasise the enactive aspect of both overt and covert (active) vision, which (from a computational perspective) renders endogenous attention an important part of ‘planning as inference’^2^. In what follows, we try to unpick the intimate relationship between attention, precision, salience and epistemic affordance^3–5^.

We live an age that offers an overwhelming access to information. However, our survival does not depend on obtaining all possible information but on seeking out that which is relevant. The relevance of information depends on one’s situation, goals or context. This means that the ‘correct’ recognition of context is essential for seeking the ‘correct’ information.

Context underwrites visual attention: in his classic study – investigating exploratory eye movements – Yarbus^6^ asked his participants to look at the same painting of a family while changing task instructions (i.e., the rule). These instructions were to either evaluate the material circumstances of the people in the picture or to guess their ages. Under the first instruction, the participants paid more attention to clothing and furniture; whereas under the second, they paid the most attention to faces. Yarbus concluded that what attracts human visual attention is information that matters^6^. This context-dependence – in visual exploration – is now a well-established phenomenon^7^. The scene context can be a background that is consistent or inconsistent with a foreground object^8–10^, or it can be defined in terms of the spatial layout of the objects^11–13^. Visual search performance has been shown to benefit from this contextual cueing as, in each case, some parts of a scene become task-relevant and contain more task-related information.

This raises the question: What is information? Shannon^14^ proposed that an outcome contains more information if it is less predictable. Itti and Baldi^15^ argue that regardless of how unexpected an outcome is, only the observations that cause a significant shift in prior beliefs (to posterior beliefs) that yield information *gain*. This notion, known as Bayesian surprise, conceptualises a unit of surprise – a “*wow*” – in terms of the difference between the prior and posterior beliefs about the world. This allows us to formulate epistemic foraging in terms of the *mutual* information between an observation, and the unobservable (hidden) states of the world that give rise to it. A new observation is presumed to be informative if the posterior distribution (about hidden states) diverges from the prior distribution. In short, observations with high Bayesian surprise attract human visual attention, but also note that, “The same data may carry different amounts of surprise for different observers, or even for the same observer taken at different times”^15^. It has also not been shown *how* Bayesian surprise can orient attention to different observations under different contexts. Here, we show, using active inference, how contextual exploration can occur – using Bayesian surprise – if beliefs about context influence beliefs about the mutual information between certain kinds of hidden state and sensory data.

Active inference is a framework that describes Bayes optimal behaviour. This framework relies upon the notion that we have an internal (generative) model encoding beliefs about how hidden states of affairs in the world ‘out there’ cause our sensations. Under active inference, exploratory and exploitative behaviours arise as a result of free energy minimisation. Variational free energy is an upper bound on the negative log Bayesian model evidence (or self-information also known as surprise). Minimising variational free energy means maximising the evidence for an internal generative model^16^. In active inference, perception and action both minimise variational free energy^17^. On this view, perception optimises beliefs about the hidden causes of sensory information, while, actions fulfil prior preferences (goals) and resolve uncertainty about the world. These actions are sampled from beliefs about policies (sequences of actions). Crucially, the agent’s beliefs about the policies it pursues are expressed in terms of the expected free energy in the future, which the agent also believes (*a priori*) it will minimize. Expected free energy comprises instrumental and epistemic value and a novelty term. Instrumental or extrinsic value is essentially the utility of a policy (i.e., the degree to which expected observations conform to prior preferences). Epistemic value is the information the agent expects to acquire about the hidden states of the environment^4,18^. Novelty is the information that can be acquired about the parameters of the generative model^19^. Formally, epistemic value is the expected Bayesian surprise or information gain afforded by a particular policy or action. It has been shown in monkeys that parietal neurons encode the expected information gain of a planned saccade, distinct from any expected reward (i.e. extrinsic value)^20^. We emphasise epistemic value in this work and show that in different contexts there may be different hidden states the agent should acquire information about.

In previous work, we have suggested that perception corresponds to inference about hidden states and attention corresponds to optimisation of the precision (i.e., confidence) afforded sensory evidence^3,21^. In this work, we consider a generative model that can adjust the precision of different sensory signals, depending on the states of the world. Active inference implies that we weight sensory inputs from different sensory channels in proportion to their precision, given our goals. In the context of rule-based or contextual exploration, this entails down-weighting the sensory precision of stimuli that are irrelevant to the context. In this way, epistemic exploration guides our sensory epithelia to relevant stimuli. Our objective here is to introduce a computational model that can selectively attend to task-relevant stimuli and acquire useful information under a particular context.

This paper comprises four sections. In the first, we describe active inference for Markov Decision processes (MDP). In the second, we introduce a selective attention task and illustrate the responses of an agent in a contextual exploration task, using an MDP formulation of active inference. We show qualitatively how selective attention and contextual exploration emerge under this scheme, where selective attention – mediated by a context-dependent sensory or likelihood precision – contextualises expected information gain. The result is behaviour that has all the hallmarks of active vision, driven by feature-based attention. This behaviour is illustrated using a minimal (colour versus shape) model that is repurposed to simulate the results reported by Yarbus. In this section, we also introduce an MDP model of a face identification task, which is used in the simulations of pathology in the subsequent section. In the third section, we apply the same principles to simulate the cardinal deficits of selective (feature-based) attention that have been reported in various conditions. Here, we focus on autism and anxiety. We conclude with a general discussion of results.

## Materials and Methods

### Active inference

A characteristic attribute of biological systems is their adaptive exchange with changing environments^22^. This adaptive exchange requires i) the change in the environment to be recognised (perception), and ii) action to be taken; in order to retain a biological system in states conducive to existence^23^. For example, living creatures can only exist in a narrow range of all possible temperatures. Another way to put this is that they must maintain a low entropy, or *surprise* (averaged over time). Active inference describes how an agent’s adaptive exchange with its environment can be described as Bayesian inference; i.e., by minimising variational free energy. Variational free energy is an upper bound on *surprise* $-\mathrm{ln}\,P(\tilde{o}|m)$. Minimising free energy therefore minimises *surprise*. Here $\tilde{o}$ represents the sequence of observations over time $\tilde{o}={[{o}_{1},{o}_{2},\mathrm{...},{o}_{T}]}^{T}$ and *m* represents the model under which *surprise* is evaluated. An agent’s perception of its environment and the actions it takes both suppress variational free energy:

For an agent to infer (perceive) the state of its environment, it requires a *generative model* that describes how observed outcomes are generated by the environment^22,24^. Variational free energy *F* is a functional of two things: the *generative model* $P(\tilde{o},x)$, and an approximate posterior distribution over the hidden causes *Q*(*x*) (Eq. 1). Rearranging this equation reveals that the variational free energy is an upper bound on *surprise* because the KL divergence in Eq. 2 can never be less than zero.

1 $$F={E}_{Q}[\,-\,{\rm{l}}{\rm{n}}\,P(\mathop{o}\limits^{ \sim },\,x)]-H[Q(x)]$$

2 $$=-\,{\rm{l}}{\rm{n}}\,P(\mathop{o}\limits^{ \sim }|m)+{D}_{KL}[Q(x)\parallel P(x|\mathop{o}\limits^{ \sim })]$$

3 $$=\,\mathop{\underbrace{{D}_{KL}[Q(x)\parallel P(x)]}}\limits_{Complexity}-\mathop{\underbrace{{E}_{Q(x)}[P(\tilde{o}|x)]}}\limits_{Accuracy}$$

Here, *x* represents hidden causes. The KL divergence is a measure of how dissimilar two probability distributions are^25,26^. More formally, it is the expected (average) log ratio of the two distributions: ${D}_{KL}[Q(x)||P(x|\tilde{o})]$ $=\sum _{x}Q(x)\mathrm{ln}(\frac{Q(x)}{P(x|\tilde{o})})$ . Two distributions are more similar if this term is small, and identical when zero. Minimising variational free energy minimises the divergence between the approximate and true posterior distributions over the hidden causes, making the former an approximation to the true distribution $Q(x)\approx P(x|\tilde{o})$. This minimises the complexity and maximises the accuracy of the observed outcomes (Eq. 3).

The process that generates the outcomes observed by the agent is called the *generative process*. The *generative model* is the agent’s internal model, used to infer the likely hidden causes of observed data – and to form beliefs about the appropriate policy (i.e., sequence of actions) to pursue. An agent who engages in active inference makes use of a prior belief that the most probable policies are those that lead to the lowest expected free energy. At each time step, the generative model is used to infer the most likely hidden states by minimising variational free energy with respect to the hidden states. This is followed by sampling an action from the beliefs about the policies. Without action selection, the agent is nothing more than an inference machine that recognises the most likely hidden states of the world. Action selection allows the agent to choose the actions that fulfil its prior preferences about the outcomes it will experience (thus minimising variational free energy), and to perform motor experiments to test perceptual hypotheses.

### Markov decision processes

**Figure 1:**
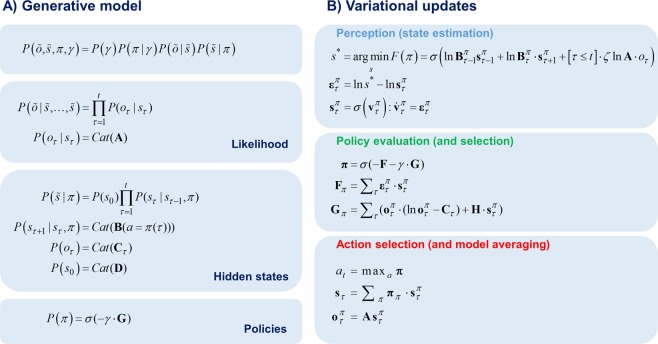
Markovian generative model (**A**) The joint probability distribution of outcomes and their hidden causes defines the generative model. Here, $\tilde{o}$ corresponds to the sequence of observations $\tilde{o}={[{o}_{1},{o}_{2},\mathrm{...},{o}_{T}]}^{T}$ over time. Similarly, $\tilde{s}$ represents the sequence of hidden states $\tilde{s}={[{s}_{1},{s}_{2},\mathrm{...},{s}_{T}]}^{T}$ over time. The generative model comprises a mapping from the hidden states to outcomes (expressed in terms of the likelihood matrix **A**) and a mapping from the previous hidden state to the next (expressed in terms of the transition matrix **B**). The notation *C at* denotes categorical distributions. The transition matrix **B** is a function of action (*a*) where action is sampled from the beliefs about the policies (*π*) for the time step *τ*. The precision term (*γ*) mediates the confidence placed in policy selection. The expected free energy (**G**) expresses how likely a policy is. The prior preference matrix (**C**) mediates how much one outcome is preferred relative to other outcomes. The initial state probability vector (**D**) encodes the probability of states at the initial time step *P*(*s*_0_). (**B)** The belief update equations shown in this panel summarise the variational message passing. Belief updates occur through three phases, namely perception, policy evaluation and action selection. In the perception phase a gradient descent on the state prediction errors ${\varepsilon }_{\tau }^{\pi }=-\,dF/d{s}_{\tau }^{\pi }$ is used to infer the most likely hidden states that generated the observed outcomes. Here s^*^ corresponds to the optimal solution to the state estimation problem, whereas ${s}_{\tau }^{\pi }$ corresponds to the current beliefs about states. The updates over the beliefs about states are iterated, using gradient descent, until the state prediction errors are minimised ${\varepsilon }_{\tau }^{\pi }\approx 0$. A softmax function is applied to ${v}_{\tau }^{\pi }=\,\mathrm{ln}\,{s}_{\tau }^{\pi }$ to obtain a probability distribution over the states. In the first equation, the precision of the likelihood matrix *ζ* mediates how much the observed outcomes influence the updates over the states (e.g. none when *ζ* = 0). In the policy evaluation phase, policies *π* are evaluated in terms of their expected free energy **G** weighted by its precision *γ* **–** and in terms of the free energy based upon previous observations **F** (please see the text for a more detailed discussion of **G** and **F)**. Finally, in the action selection phase an action is sampled from the most likely policy **π** (i.e. policy with the highest posterior probability). The subsequent equation shows that the beliefs about the states ${s}_{\tau }$ are obtained by taking the expectation of beliefs about the states expected under policies ${s}_{\tau }^{\pi }$ with respect to the beliefs about the policies **π**. The final equation shows that the outcomes expected under a policy ${o}_{\tau }^{\pi }$ at time step *τ* are obtained by taking the expectation of the likelihood matrix **A** with respect to the beliefs about the hidden states under policies ${s}_{\tau }^{\pi }$.

Markov decision process (MDP) models use a discrete state space to describe how events evolve through time, giving rise to categorical outcomes at each (discrete) time point^27^. The MDP generative model comprises several matrices and vectors that define the probabilistic structure of the world. The likelihood matrix ***A*** is a mapping from hidden states to outcomes, indicating which outcomes are more likely under different hidden states. The transition matrix *B* defines the transition probabilities between different hidden states. The initial state probability vector ***D*** indicates which states the agent believes are more likely initially. The generative model also embodies an agent’s prior beliefs about outcomes in the prior preference matrix ***C***, which defines how much one outcome is preferred compared to another (see Fig. 1A for the MDP generative model).

Crucially, in active inference the agent has some control over parts of the environment. This means that the agent can control the transitions between some of the hidden states, through actions sampled from beliefs about policies *π*. This means that the observed sensory input depends on the hidden states that can be controlled through actions. Prior beliefs about policies (or sequences of actions) are defined in terms of an expected free energy of future outcomes. This way the agent plans future actions such that they minimise the expected free energy in the future. The expected free energy is expressed as:

4 $$\begin{array}{rcl}G(\pi ) & = & \sum _{\tau }G(\pi ,\tau )\\ G(\pi ,\tau ) & = & {E}_{\tilde{Q}}[\mathrm{ln}\,Q(A,{s}_{\tau }|\pi )-\,\mathrm{ln}\,P(A,{s}_{\tau },{o}_{\tau }|\tilde{o},\pi )]\\  & = & {E}_{\tilde{Q}}[\mathrm{ln}\,Q(A)+\,\mathrm{ln}\,Q({s}_{\tau }|\pi )-\,\mathrm{ln}\,P(A|{s}_{\tau },{o}_{\tau },\tilde{o},\pi )-\,\mathrm{ln}\,P({s}_{\tau }|{o}_{\tau },\tilde{o},\pi )-\,\mathrm{ln}\,P({o}_{\tau })]\\  & \approx  & {E}_{\tilde{Q}}[\mathrm{ln}\,Q(A)+\,\mathrm{ln}\,Q({s}_{\tau }|\pi )-\,\mathrm{ln}\,Q(A|{s}_{\tau },{o}_{\tau },\pi )-\,\mathrm{ln}\,Q({s}_{\tau }|{o}_{\tau },\pi )-\,\mathrm{ln}\,P({o}_{\tau })]\\  & = & -\mathop{\underbrace{{E}_{\tilde{Q}}[\mathrm{ln}\,Q(A|{s}_{\tau },{o}_{\tau },\pi )-\,\mathrm{ln}\,Q(A)]}}\limits_{{novelty}}-\mathop{\underbrace{{E}_{\tilde{Q}}[\mathrm{ln}\,Q({s}_{\tau }|{o}_{\tau },\pi )-\,\mathrm{ln}\,Q({s}_{\tau }|\pi )]}}\limits_{{epistemicvalue}}-\mathop{\underbrace{{E}_{\tilde{Q}}[\mathrm{ln}\,P({o}_{\tau })]}}\limits_{{extrinsicvalue}}\end{array}$$

where $$\tilde{Q}=Q({o}_{\tau },{s}_{\tau }|\pi )=P({o}_{\tau }|{s}_{\tau })Q({s}_{\tau }|\pi )\approx P({o}_{\tau },{s}_{\tau }|\tilde{o},\pi )$$.

Expected free energy comprises three terms; namely, novelty, epistemic and extrinsic value (see Eq. 4). Extrinsic value is the expected utility defined in terms of prior preferences over the outcomes^28^. Epistemic value is the information that can be acquired by reducing uncertainty about the hidden causes in the environment^4^. Novelty is the information that can be gained about the parameters of the generative model^19^. A policy carries a high novelty value if it affords the opportunity to reduce uncertainty about contingencies (encoded in the likelihood matrix, transition matrix and initial state probabilities) that underwrite how outcomes are generated. A policy has epistemic value (i.e., has epistemic affordance), if the outcomes *o*_*τ*_ expected under that policy resolve uncertainty about hidden states *s*_*τ*_ – this is sometimes referred to as salience. Novelty and salience drive exploration, whereas the extrinsic component of the expected free energy drive exploitation. The resolution of the exploration-exploitation dilemma thus rests upon the balance between the novelty, epistemic and extrinsic value of a policy. Crucially, these components are all expressed in same units (log-probabilities) and therefore share a common currency or metric.

### Belief updates and message passing

Under active inference, perception arises from minimising variational free energy with respect to beliefs about hidden variables. Mathematically, this is implemented via a gradient descent on the variational free energy for each hidden variable. The resulting belief update equations show how message passing occurs under this scheme (see Fig. 1B).

Taking the gradient of variational free energy, with respect to the hidden states, after observing a new outcome gives the optimal solution to state estimation denoted by s^*^ (state estimation: first equation). The difference between s^*^ and the current beliefs about the hidden states ${s}_{\tau }^{\pi }$ generates a state prediction error ${\varepsilon }_{\tau }^{\pi }$ (second equation). A gradient descent on state prediction errors is used to infer the most likely hidden states (third equation). Here, *τ* represents the time steps from 1 to *t* + 1. When *τ* ≤ *t*, the term in brackets (first equation) returns 1 and otherwise it returns 0. This means that the inference about the hidden states at the current time step *t* depends on the observed outcomes from time step 1 to *t*, which allows for evidence accumulation over time. When *τ* > *t* beliefs about the hidden states do not depend on the outcomes as the outcomes have not been observed yet. This means that beliefs about the hidden states at *τ* > t depend only upon beliefs about hidden states in the previous ${s}_{\tau -1}^{\pi }$ and next ${s}_{\tau +1}^{\pi }$ time steps.

Beliefs about the inferred hidden states are projected into the future to form expectations about the most likely observations in the future under different policies. These expectations are used to compute the probability distribution over policies *π* (policy evaluation: first equation) such that the most likely policy has the smallest free energy **F** (second equation) and expected free energy **G** (third equation). **F** and **G** are vectors with elements corresponding to each policy. The free energy **F**_*π*_ under a policy is a function of the state prediction errors under that policy and the beliefs about states under that policy. The expected free energy G_*π*_ is expressed in terms of risk and ambiguity. Risk is the expected divergence from preferred outcomes and expressed as the (expected) difference between (log) expected outcomes $\mathrm{ln}\,{{\bf{o}}}_{\tau }^{\pi }$ and preferred (log) outcomes **C**_*τ*_ expected under beliefs about future outcomes ${{\bf{o}}}_{\tau }^{\pi }\cdot (\mathrm{ln}\,{{\bf{o}}}_{\tau }^{\pi }-{{\bf{C}}}_{\tau })$. Ambiguity is the expected uncertainty in the mapping from hidden states to observations expected under beliefs about the hidden states ${\rm{H}}\cdot {s}_{\tau }^{\pi }$, where H is the entropy of outcomes under all possible combinations of hidden states.

Action selection involves sampling an action *a*_*t*_ from the most likely policies (action selection: first equation), where ***π*** corresponds to the beliefs about the policies. The expected states and outcomes are acquired by taking Bayesian model averages of the states (second equation) and outcomes (third equation) expected under each policy. Once an action is selected, the environment will generate a new outcome that can be fed back to the generative model and thus the perception and action cycle begins again.

## Visual Attention Tasks

### Colour/Shape task

**Figure 2:**
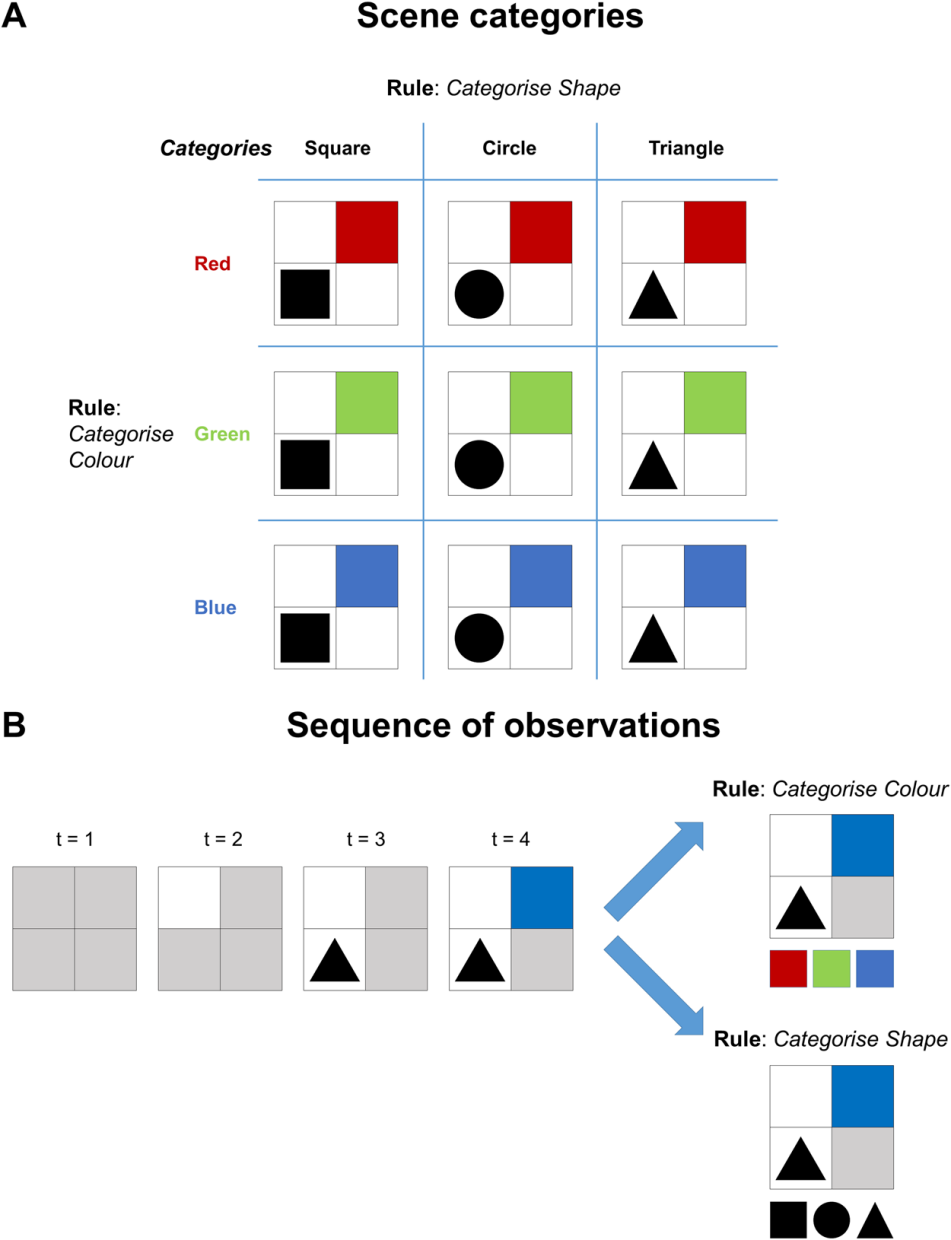
Contextual exploration task (**A**) A scene can be categorised in two ways, either by its colour or its shape. The categories are shown on the left and on top when the rule (context) is to categorise the scene in terms of its colour and shape, respectively. (**B)** The sequence of observations illustrates exploration of an exemplar scene. In the beginning, each quadrant is greyed out (t = 1). Attending to (i.e. looking at) each quadrant reveals its content. In this case, the order of explored locations is top left (t = 2), bottom left (t = 3) and top right quadrants (t = 4).

The colour/shape task is performed on a two-by-two grid scene, whose quadrants are initially masked. Attending to a quadrant unmasks the object in that quadrant (see Fig. 2B). In this task, certain objects are associated with certain contexts. These contexts can be seen as rules that state what information should be sought, very much like the instructions in Yarbus’ experiment^6^. The goal in this task is to categorise the scene that is being explored.

A scene can be categorised either in terms of its *colour* or its *shape*. These attributes constitute the hidden states that the agent must infer. The agent knows the categorisation rule before performing the task: the colour category is determined by the colour in the top right quadrant (the top right can be red, green or blue). The category of the scene is red, green or blue if the object in the top right quadrant is red, green or blue, respectively; given that the rule is *categorise colour*. The shape category of the scene is square, circle or triangle, if the object in the bottom left quadrant is a square, circle or triangle, respectively; given that the rule is *categorise shape* (see Fig. 2A). Beliefs about the category of the scene (i.e., hidden states) are reported by looking at one of three choice locations at the bottom of the scene. These choice locations either correspond to *colour* or *shape* categories depending on the *rule* (see the rightmost panels in Fig. 2B). Upon declaring a categorisation, the agent receives feedback of *right* or *wrong*.

### MDP model

In this MDP model, we considered four sets of hidden states, namely *Rule*, *Where*, *Category: colours* and *Category: shapes*. The first set of hidden states *Rule* defines the context in which the scene is categorised. A scene can be categorised in two ways, either in terms of its *colour* or its *shape*, depending upon the rule. The second set of hidden states *Where* corresponds to the locations in the scene. There are eight locations in this task: central fixation (location 1), the four quadrants (locations 2–5) and three choice locations (locations 6–8) at the bottom. The choice locations are associated with the categories *red*, *green* and *blue* when the rule is *categorise colour*, and *square*, *circle* and *triangle* when the rule is *categorise shape*. The third set of hidden states *Category: colours* controls what colours will appear on the top right quadrant under the colour categories *red*, *green* and *blue*; e.g., if the colour category is *blue* then the colour *blue* will be in this location. The fourth set of hidden states *Category: shapes* determines which shape will be in the bottom left quadrant under the shape categories *square*, *circle* and *triangle*.

**Figure 3:**
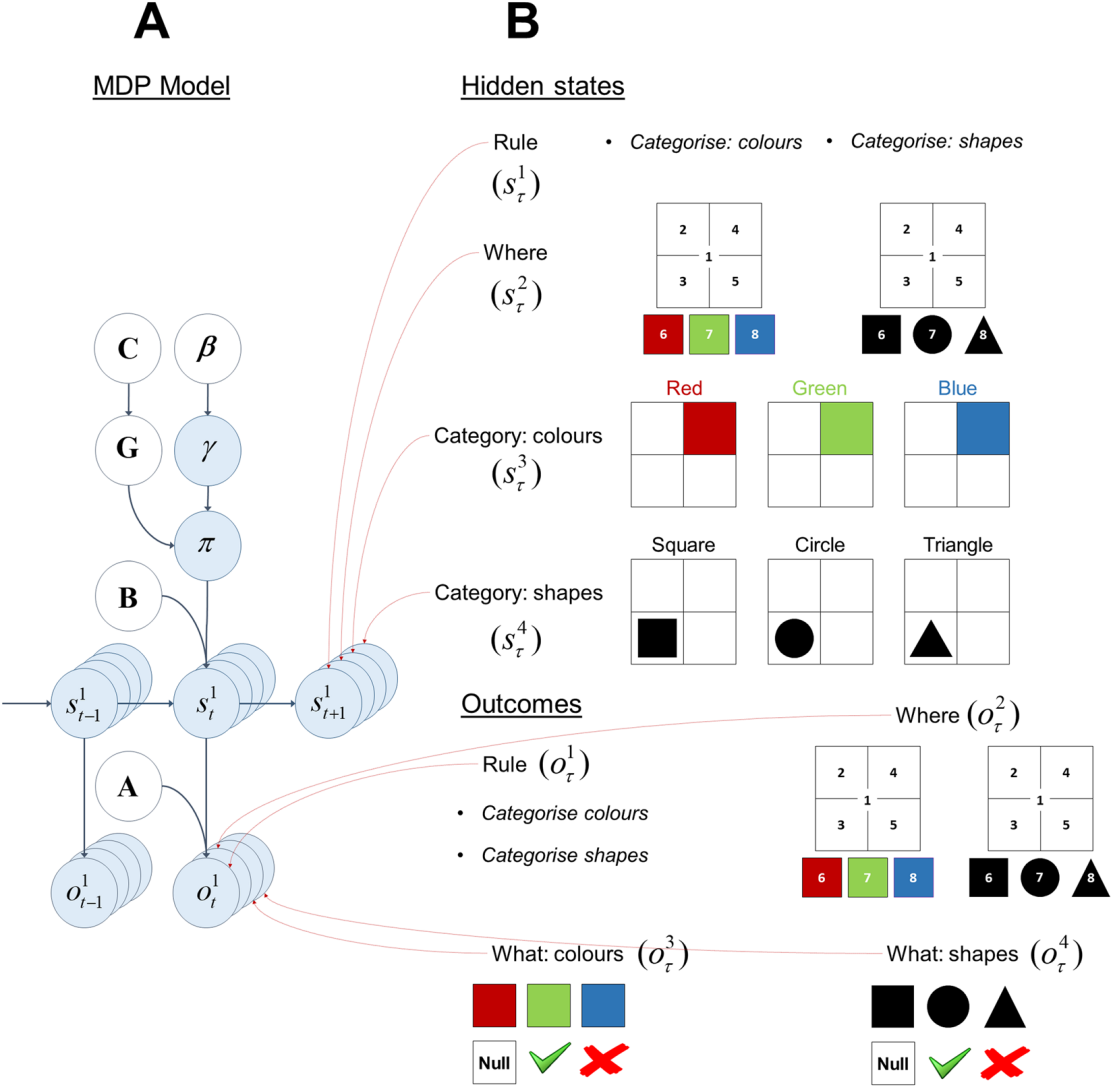
Structure of the generative model – colour/shape task (**A**) This panel shows the graphical representation of the MDP model and the conditional dependencies among the terms in the model. The structure of the environment is expressed in terms of the transition and likelihood matrices. The likelihood matrix (**A**) is a mapping from the hidden states (*s*_*t*_) to the outcomes (*o*_*t*_). The state transitions are mediated by the transition matrix (**B**) which expresses how likely the current state (*s*_*t*_) is given the previous state (*s*_*t*−1_). Crucially, the transition matrix is a function of action which can be sampled from the beliefs about the policies. The beliefs about the policies (*π*) depend on the expected free energy (**G**) and the precision of policy selection (***γ***). The expected free energy comprises extrinsic and epistemic values. Extrinsic value is a function of the prior preference matrix (**C**) which encodes how much one outcome is expected relative to another. Precision of policy selection (***γ***) is a function of the temperature term (***β***). The smaller the temperature the more deterministic the policy selection becomes. (**B)** This panel shows the four sets of hidden states and outcome modalities in the colour/shape task. There are four sets of hidden states, namely *Rule*, *Where*, *Category: colours* and *Category: shapes*. There are four outcome modalities, namely *Rule*, *Where*, *What: colours* and *What: shapes*.

We considered four outcome modalities, namely *Rule*, *Where*, *What: colours* and *What: shapes*. The first outcome modality *Rule* unambiguously cues the context, either categorise in terms of *colour* or *shape*. The second outcome modality *Where* signals the sampled location in the scene (one of eight locations). This can be thought of as a proprioceptive signal. The third outcome modality *What: colours* signals which colour is observed in the sampled location. It can be *red*, *green*, *blue* or *null* (no colour). The fourth outcome modality *What: shapes* signals which shape is observed in the sampled location. It can be *square*, *circle*, *triangle* or *null* (no shape). Under both *What: colours* and *What: shapes* modalities there are two additional feedback outcomes, *right* and *wrong* (see the green tick and red cross in Fig. 3). An agent can report its beliefs about the category of the scene by choosing one of the three choice locations associated with the categories under the rules *categorise colour* or *categorise shape* and obtain feedback about whether its choice was *right* or *wrong*. See Fig. 3 for the hidden states and outcome modalities.

**Figure 4:**
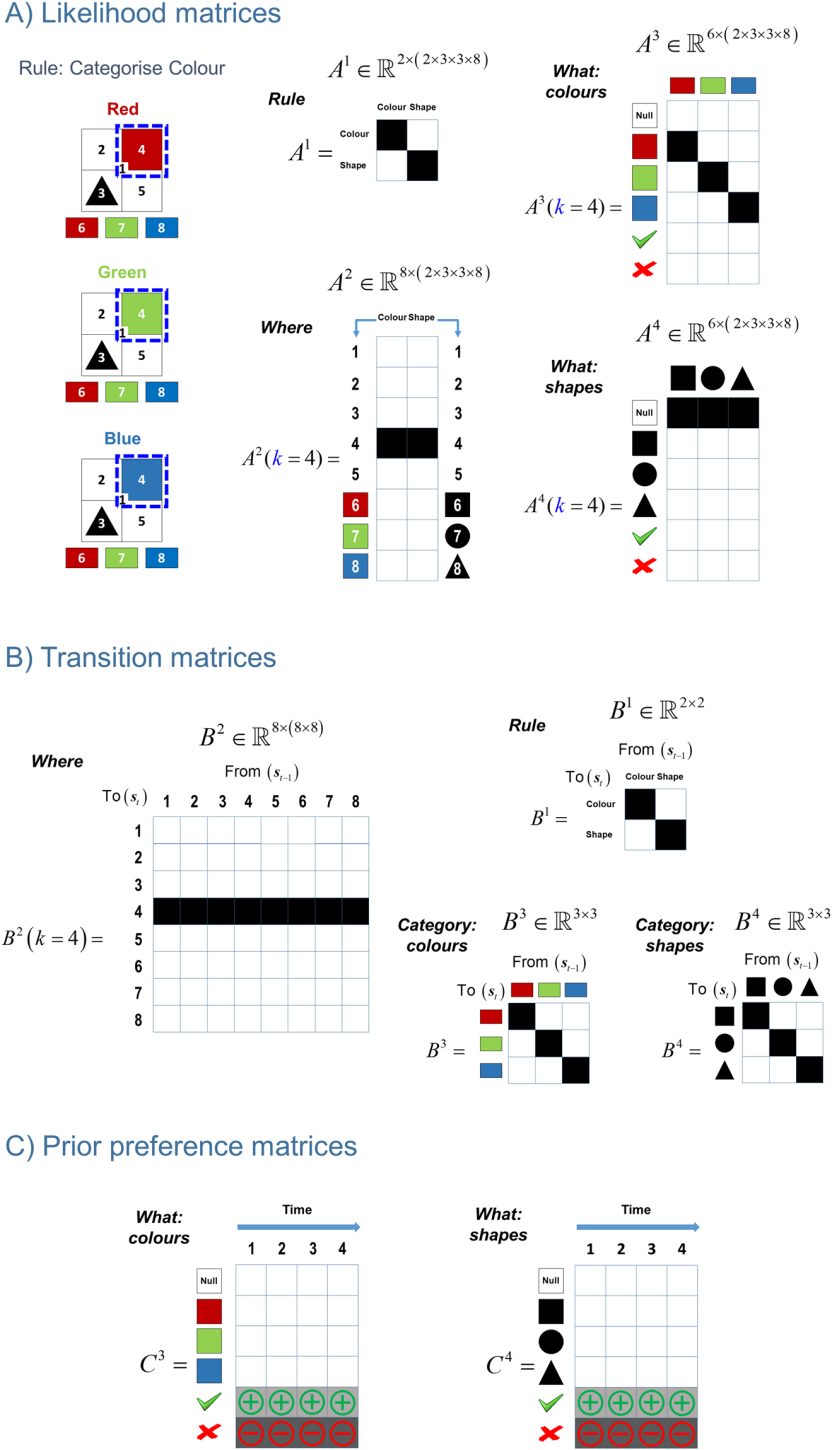
ABC of generative model. This figure shows the likelihood, transition and prior preference matrices used in the colour/shape task. (**A**) The colour category of the scenes shown on the left is determined purely by the colour in location 4 (top right quadrant). The panels on the right show the likelihood (**A**) matrices for location *k* = 4. The likelihood matrices encode the probability of outcomes (*o*_*t*_) given the hidden states (*s*_*t*_). The first likelihood matrix *A*^1^ (*Rule*) signals what the rule is, either categorise *colour* or *shape*. The second likelihood matrix *A*^2^ (*Where*) signals the sampled location on the scene, one of *eight* locations. The third likelihood matrix *A*^3^ (*What: colours*) encode the probability of colours *red*, *green* and *blue* under different colour categories *red*, *green* and *blue*. The final likelihood matrix *A*^4^ (*What: shapes*) encode the probability of shapes *square*, *circle* and *triangle* under different shape categories *square*, *circle* and *triangle*. Because the colour and shape are separate modalities, the probability of colour and shape objects are encoded by separate likelihood matrices *A*^3^ and *A*^4^. The likelihood matrix under the colour modality for location 4 *A*^3^(k = 4) shows that the colour category of a scene is purely determined by the colours in this location; however, under the shape modality *A*^4^(k = 4) the object in this location does not provide any information about the shape category, i.e. *null*. (**B)** This panel shows the transition matrices. All the transition matrices are identity matrices except for the action dependent transition matrix *B*^2^, which encodes the most likely location to be sampled as a function of action, e.g. *B*^2^(*k* = 4) shows that under action **4**, the top right quadrant is the most likely location to be sampled at the next time step. The identity transition matrices *B*^1^ (*Rule*), *B*^3^ (*Category: colours*) and *B*^4^ (*Category: shapes*) express the fact that the *rule* and the *colour* and *shape* objects do not change in the course of a trial. **C)** The prior preference matrices are shown in this panel. The prior preference matrices encode how much one outcome is preferred relative to other outcomes as a function of time. The only definitive preferences are defined over the columns of *C*^3^ (*What: colours*) and *C*^4^ (*What: shapes*). Under both *C*^3^ and *C*^4^ the utility of making a right categorisation and wrong categorisation is +2 and −4 natural units, respectively. With these utilities (i.e. log odds) the agent expects to categorise a scene correctly, while avoiding an incorrect categorisation.

In this setup, the *Rule* and *Where* hidden states directly map to the *Rule* and *Where* outcomes. The *Rule* outcome is used to update the agent’s beliefs about the *Rule* hidden state. In this sense, the *Rule* hidden state may be considered as an explanatory variable, used to account for the sensory data cueing the rule. Crucially, this explanation has consequences for the interpretation of the other sensory outcomes: precise beliefs about the *Rule* allow the agent to increase the precision of objects that are task relevant, while decreasing the precision of those that are not. This enables the agent to attend selectively to objects in the scene. The hidden states *Category: colours* and *Category: shapes* map onto *What: colours* and *What: shapes* objects as a function of *Where* and *Rule* hidden states; e.g., sampling location 8 when the rule is *categorise colours* would generate a *right* feedback, if the scene category is *blue*. All the transition matrices are identity matrices except for the action dependent *Where* transition matrix. The identity matrices indicate that the rule and the scene category do not change during the course of a trial. The action dependent *where* transition matrix specifies that the agent would look at the location indicated by the action, e.g. if the sampled action is 4 then the agent would go to the top right location. In this setup, we defined prior preferences over *right* (utility or relative log probability of 2 nats) and *wrong* (utility of −4) outcomes under both *What: colours* and *What: shapes* modalities. With these utilities the agent avoids categorising the scene prematurely and categorises only once it has accumulated sufficient evidence. See Fig. 4 for the likelihood, transition and prior preference matrices.

### Simulations

In active inference, exploration of a scene would usually continue until all uncertainty about the hidden states of the world (here, colour and shape categories) is resolved. However, rule-based exploration requires one to resolve uncertainty only about the relevant hidden states. There is no imperative to resolve uncertainty about the *shape* category when the rule is *categorise colour*. Here, we show how this selective, context-sensitive epistemic foraging can arise as a function of selective attention.

Technically, the information gained from observing a stimulus depends on the precision *ζ* of the likelihood mapping between that stimulus and an unknown hidden state; e.g., the degree to which seeing ‘blue’ means the scene must be a ‘blue’ category. By reducing the precision of the task-irrelevant likelihood, an agent can reduce the expected information gained from observing task-irrelevant objects, and thus ignore or ‘attend away’ from them.

**Figure 5:**
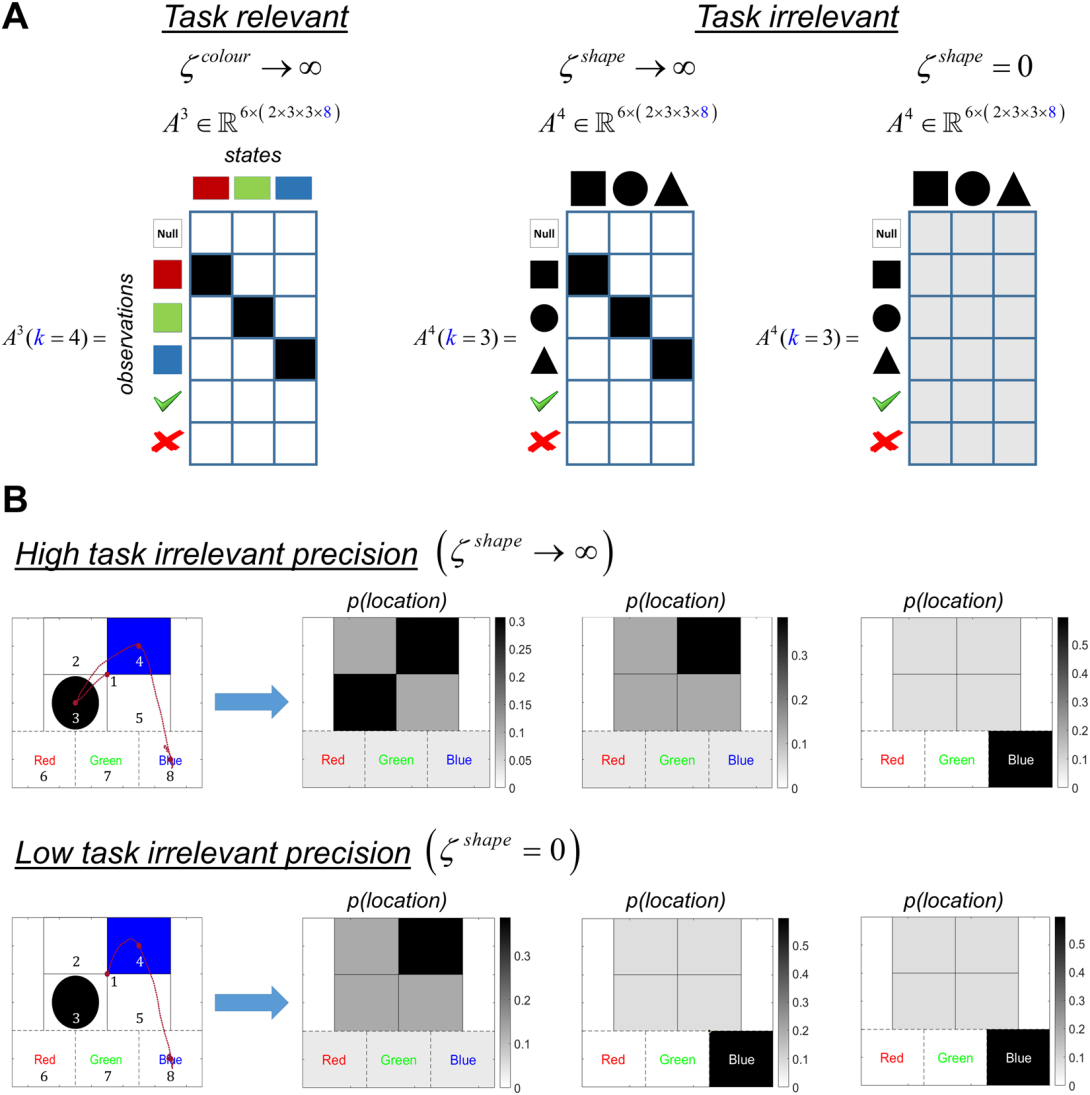
Contextual exploration In this figure, the rule is to categorise a scene in terms of its *colour*. The objects that resolve uncertainty about the *colour* category are task-relevant, and the objects that resolve uncertainty about the *shape* category are task-irrelevant. (**A)** The likelihood matrix on the left shows the mapping between the task-relevant objects and categories (in this case *colour* objects and categories), under high precision *ζ*^*colour*^ → ∞. The likelihood matrices in the middle and right panels show how the mapping between the task-irrelevant objects and the categories (in this case *shape* objects and categories) change under two levels of precision *ζ*^*shape*^. Under a high precision *ζ*^*shape*^ → ∞ this mapping is very precise, however under a low precision *ζ*^*shape*^ = 0 it becomes very ambiguous. When this mapping is imprecise the agent no longer acts to resolve uncertainty about the task-irrelevant category. The task-relevant and irrelevant likelihood matrices are illustrated for locations 3 and 4, because these are the only locations that hold the objects that resolve uncertainty about the *shape* and *colour* categories, respectively. The precision term *ζ*^*colour*^ is used to change the mapping between *colour* objects and categories in the same way when the rule is *categorise shape*. (**B)** The upper and lower left panels show how the exploratory behaviour changes under two levels of task-irrelevant precisions *ζ*^*shape*^ → ∞ and *ζ*^*shape*^ = 0, while keeping task-relevant precision high *ζ*^*colour*^ → ∞. The subsequent panels show how likely an agent is to sample a location during the course of a trial, expressed in terms of prior probabilities for each policy. At the beginning of each trial, the agent fixates at the centre of the screen (location 1). Under high task-irrelevant *ζ*^*shape*^ → ∞ and task-relevant *ζ*^*colour*^ → ∞ precisions, the agent finds that it is equally likely to attend to the task-relevant *colour* objects in location 4 as the task-irrelevant *shape* objects in location 3, in the beginning. The agent first attends to location 3 where it finds a *circle* and then attends to location 4, where it finds the colour *blue*. Subsequently the scene is categorised as *blue*. Under a low task-irrelevant precision *ζ*^*shape*^ = 0 and high task-relevant precision *ζ*^*colour*^ → ∞ the agent infers that the only location that matters is location 4, which holds the task-relevant *colour* objects. In the next time step it attends to location 4 and finds the colour *blue* and subsequently categorises the scene as *blue*.

An example is provided in Fig. 5, where the rule is to categorise the scene in terms of its colour. Under this rule, colour objects are task-relevant and shape objects are task-irrelevant. Thus, when performing the categorisation task, an agent would only attend to the colour objects if *ζ*^*colour*^ is maximised (Fig. 5A, left panel) and *ζ*^*shape*^ is minimised, i.e. *ζ*^*shape*^ = 0 (Fig. 5A, right panel). If *ζ*^*shape*^ precision is maximised (i.e., *ζ*^*shape*^ → ∞, see Fig. 5A, middle panel), the agent becomes more likely to attend to task-irrelevant objects.

Note that the agent’s beliefs about the likelihood mapping and the mapping in the real world may not be the same: i.e., the generative model (internal beliefs) and generative (real-world) process may be different. In the right panel of Fig. 5A, the agent believes that there is an imprecise mapping between *shape* categories and objects (generative model) but this mapping is very precise in the process that generates outcomes (generative process). In fact, the middle and right panels of Fig. 5A illustrate cases when the generative model and generative process are identical and different, respectively. These panels show how varying *ζ*^*shape*^ changes the mapping between the task-irrelevant *shape* category and objects when the rule is *categorise colour*. The mapping between *colour* category and objects are changed in the same way using *ζ*^*colour*^ when the rule is *categorise shape*.

The upper and lower left panels of Fig. 5B show the quadrants that the agent attended to in the course of a trial under high *ζ*^*shape*^ → ∞ and low *ζ*^*shape*^ = 0 levels of task-irrelevant precision, while keeping the task-relevant precision high *ζ*^*colour*^ → ∞ for both trials. The heat maps in the right panels show how likely the agent is to attend to a particular location in the scene, expressed in terms of a softmax function of expected free energy under eight policies (i.e. visiting one of the eight locations in the scene). On the trials shown in Fig. 5B, the rule is to categorise the scene in terms of its *colour*. When the agent believes that it can acquire information about the task-irrelevant *shape* category (i.e. *ζ*^*shape*^ → ∞; upper panel), it finds that it is equally likely to attend to the top right (colour) and bottom left (shape) quadrants at *t* = 1, even though the only object that can resolve uncertainty about the *colour* category is in the top right quadrant. The agent chooses between the two randomly – in this case, the bottom left (shape) quadrant – and only then attends to the top right (colour) quadrant at *t* = 2, successfully categorising the scene as *blue* at *t* = 3. Conversely, when the agent does not believe that it can resolve uncertainty about the task-irrelevant *shape* category *ζ*^*shape*^ = 0 (lower panel), it ignores the bottom left (shape) quadrant and categorises the scene as *blue* one timestep earlier.

Epistemic exploration seeks out the information that can be acquired about an environment. However more often than not, the information out there is not useful to the task at hand. In the next section we show that attentional mechanisms need to be in play for contextual exploration to occur and how information that is task-relevant can be acquired.

### Contextualising epistemic exploration

In the model described above, the uncertainty that can be resolved through exploration is about the scene category in terms of its *colour* and *shape*. Epistemic exploration favours saccades to the locations that offer information about *colour* and *shape* categories of the scene regardless of what the *Rule* is. Rule-based (contextual) exploration requires an agent’s attention to be directed such that only relevant information under a context matters. The most salient actions are then those that yield observations (in this case *colour* and *shape* modalities) that are generated by hidden states (objects under *colour* and *shape* categories) with a high fidelity (precision). Here, we show that beliefs about the uncertainty in the mapping from the hidden states of the world *s*_*t*_ to sensory observations *o*_*t*_ can modulate the salience associated with saccades to each location^5^.

In the colour/shape task, the precision of the sensory signals is modulated as a function of the *Rule* hidden state dimension in the generative model. This works such that when the *Rule* hidden state is *categorise: colour* the sensory precision of the *shape* objects becomes low while the sensory precision of the *colour* objects becomes very high, and vice versa for hidden state *categorise: shape*. This can be expressed formally as follows:

5 $${A}_{nijkl}^{m}=P({o}^{m}=n|{s}^{1}=i,{s}^{2}=j,{s}^{3}=k,{s}^{4}=l)$$

6 $$P({o}^{m}=n|{s}^{1}=i,{s}^{2}=j,{s}^{3}=k,{s}^{4}=l)=\sigma ({\zeta }_{i}^{m}\,\mathrm{ln}\,{\bar{A}}_{nijkl}^{m})$$

7 $$\zeta =\begin{array}{c}{\boldsymbol{m}}\,\,\,\,\end{array}\begin{array}{c}Rule\\ Where\\ What:Colour\\ What:Shape\end{array}\mathop{\mathop{[\begin{array}{cc}\,\,\,{\rm{\infty }} & \,\,\,\,\,\,\,\,\,\,\,\,\,{\rm{\infty }}\,\,\,\\ \,\,\,{\rm{\infty }} & \,\,\,\,\,\,\,\,\,\,\,\,\,{\rm{\infty }}\,\,\,\\ \,\,\,{\rm{\infty }} & \,\,\,\,\,\,\,\,\,\,\,\,\,z\,\,\,\\ \,\,\,z & \,\,\,\,\,\,\,\,\,\,\,\,\,{\rm{\infty }}\,\,\,\end{array}]}\limits^{\begin{array}{cc}Categorise & Categorise\\ colours & shapes\\ \end{array}}}\limits^{{\boldsymbol{i}}}$$

Equation 5 expresses the likelihood of the outcome *o*^*m*^ = *n* in the *generative process* given the hidden states *s*^1^ = *i*, *s*^2^ = *j*, *s*^3^ = *k* and *s*^4^ = *l* with *m* ∈ *M*, *n* ∈ *N*, *i* ∈ *I*, *j* ∈ *J*, *k* ∈ *K*, *l* ∈ *L* where *M* is the number of different outcome modalities (*Rule*, *Where*, *What: colours* and *What: shapes*), *N* is the number of outcomes in an outcome modality (e.g. under the *What: colours* modality red, green and blue colours) and,

$$\begin{array}{ccc}I & = & \{categorise\,colour,\,categorise\,shapes\},\\ J & = & \{location\,1,\ldots ,\,location\,8\},\\ K & = & \{red,\,green,\,blue\}\,{\rm{a}}{\rm{n}}{\rm{d}}\,L=\{square,\,circle,\,triangle\}\end{array}$$

are the number of states under different hidden state dimensions (e.g. under the first hidden state dimension *s*^1^, *Rule* states *categorise: colours* and *categorise: shapes*).

Equation 6 expresses the likelihood of the same outcome in Eq. 5 but this time for the *generative model*. This likelihood mapping is subject to the precision terms ζ. The precision term ${\zeta }_{i}^{m}$ is applied to the logarithm of the likelihood matrix for the *m*-th outcome modality *A*^*m*^ given the *i*-th level of the first hidden state *s*^1^ = *i*. Finally, a softmax function is applied to the resulting term to normalise the columns of the likelihood matrix to the range of probabilities.

The matrix in Eq. 7 is a precision matrix, which shows the values of the precision terms ${\zeta }_{i}^{m}$ for different outcome modalities *m* and different levels of the first hidden state dimension *s*^1^ = *i* which specifies what the rule is (*categorise: colour* or *categorise: shape*). This matrix shows that the precision term ${\zeta }_{i}^{m}$ is infinitely large under the modalities *Rule* and *Where* for $\forall \,i$, which means that there is a deterministic mapping to the outcomes under these two modalities. The crucial manipulation is implemented under *What: colours* and *What: shapes* modalities. When the first hidden state dimension *s*^1^ is on the $i=categorise:\,\,colours$ level (see the first column of the precision matrix) there is a very precise mapping to the colour objects (${\zeta }_{i}^{m}=\infty$) under the *What: colours* modality. With this precise mapping, the agent thinks that it can resolve uncertainty about the *Category: colours* hidden state (see the left panel of Fig. 5A). The precision of the mapping from $i=categorise:\,\,colours$ to shape objects under *What: shapes* modality is expressed as a function of *z*. When *z* = 0 the mapping to the shape objects become very imprecise, which means the agent believes it cannot resolve uncertainty about *Category: shapes* hidden state. Therefore, the agent’s attention is directed only to task-relevant objects; namely, colour objects (see the right panel of Fig. 5A). When *z* → ∞ the mapping to the shape objects are very precise which means that the agent’s attention would be divided between two different outcome modalities, namely *What: colours* and *What: shapes*, that could resolve uncertainty about the two hidden states, namely *Category: colours* (task-relevant) and *Category: shapes* (task-irrelevant) categories (see the middle panel of Fig. 5A). A similar formulation is shown in the second column of the precision matrix when the rule is *categorise: shapes*.

State prediction errors are used to infer the most likely hidden states of the world in the perception phase of the variational updates (see Fig. 1B). Notice that the precision term *ζ* multiplies the logarithm of the likelihood matrix ln *A*, in the first equation under perception. This shows that when sensory precision is very low *ζ* = 0 the observation *o*_*τ*_ does not contribute to state prediction errors in the second equation and does not influence inference implicit in the variational updates.

## Yarbus’ Task

The same principles and model can be applied to Yarbus’ paradigm by extending and renaming the hidden states. In what follows, we describe how the above formulation reproduces both the visual exploration during free searches – and the selective attention to informative cues elicited in the original Yarbus paradigm: as in the colour/shape model, the scene can be explored in two distinct ways, depending on task instructions. When the instruction is *estimate the family’s material circumstances*, the only objects that matter are the furniture and people’s clothing, whereas when the instruction is *give the ages of the people*, faces hold the most information.

**Figure 6:**
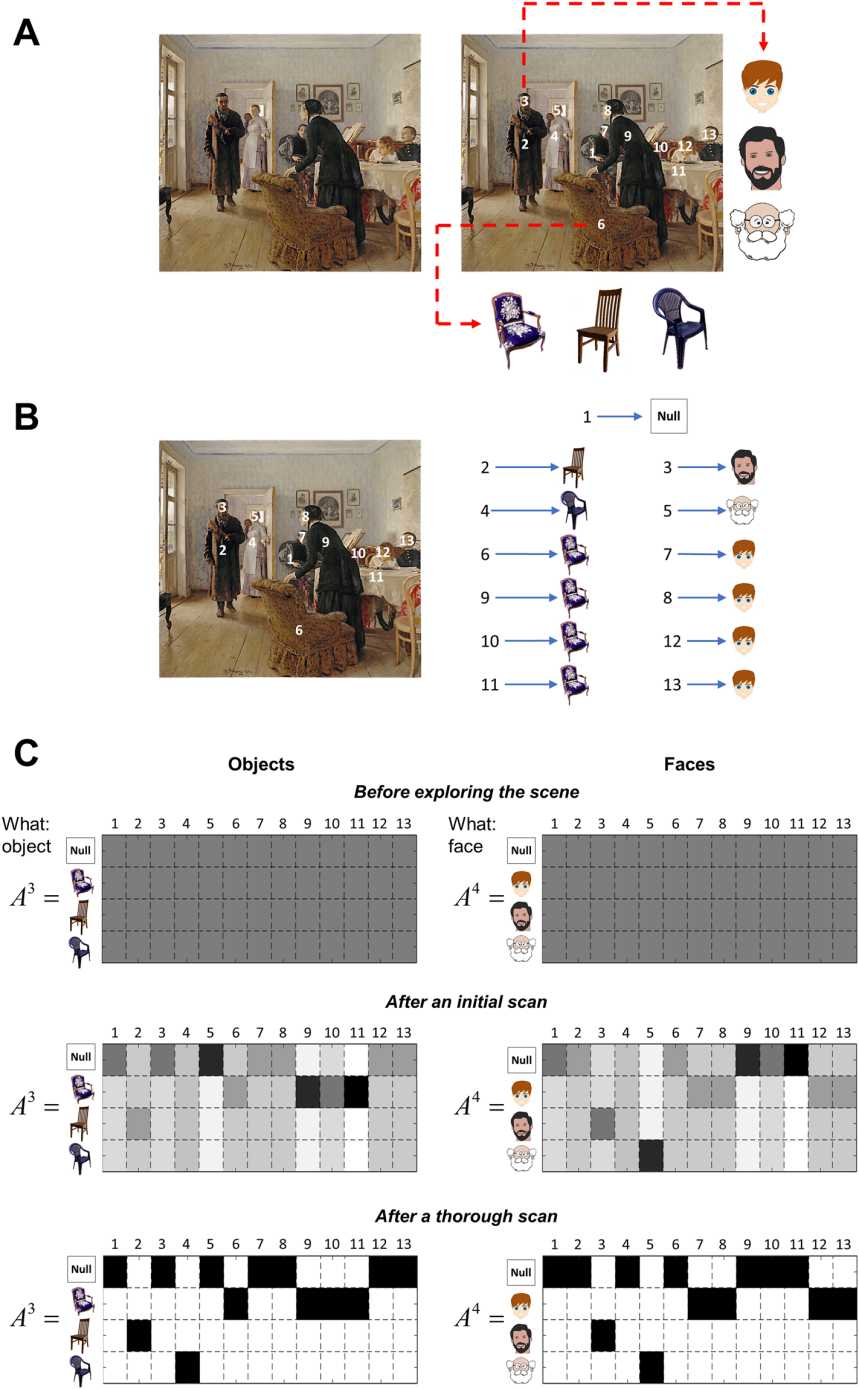
Learning the locations of the features in Yarbus’ task. **(A**) The painting *An Unexpected Visitor* by Ilya Repin is shown on the left. The right panel highlights 13 locations that can furnish information about either *material circumstances* of the family or *the ages* of the people in the painting. The first location is the centre of the scene. Furniture and clothing appear at locations 2, 4, 6, 9, 10 and 11. Faces appear at locations 3, 5, 7, 8, 12 and 13. For illustrative purposes, location 3 has been chosen to show that one can see *young*, *middle aged* or *old* faces in locations a face can appear. Location 6 has been chosen to show that one can see an *antique*, a *modest* or a *common chair* in locations a piece of furniture (or man’s/woman’s clothing) can appear. (**B)** This panel shows a scene in which locations 2 and 4 hold a modest and a common chair, respectively, whereas locations 6, 9, 10 and 11 locate antique chairs. Similarly, locations 3 and 5 hold a middle aged and an old face, respectively, whereas locations 7, 8, 12 and 13 locate young faces. (**C**) Agents learn the locations of the features shown in panel B by accumulating counts for each state-outcome pair, where the novelty term (see Eq. 4.) drives behaviour such that exposure to these novel combinations is assured. This enables the agent to learn the (likelihood) mapping from locations to objects *A*^3^ and faces *A*^4^ in the course of free visual exploration. The likelihood matrices – on the top – show that the agent initially has no knowledge of which features are located where. The matrices in the middle show that the agent starts to learn the locations of the objects and faces after an initial scan of the scene. The matrices at the bottom show that after a thorough exploration of the scene, the agent has precise knowledge of the object locations. The painting Unexpected Visitors by Ilya Repin has been downloaded from https://commons.wikimedia.org/w/index.php?title=File:Ilya_Repin_Unexpected_visitors.jpg ^71^. The cartoon faces and the pictures of the chairs in this figure have been downloaded from https://pixabay.com/.

The subjects in Yarbus’ study knew where to expect certain objects in the painting because they were asked to explore the scene freely, before exploring the same scene under different instructions. In this setting, one expects some objects to appear in certain locations^29^: for example, furniture and faces tend to be located at different heights, and different positions relative to other objects. In our model, these locations are highlighted with numbers between 1 and 13, where location 1 corresponds to the centre of the scene (see the right panel of Fig. 6A). The furniture and clothes appear in locations 2, 4, 6, 9, 10, 11, whereas the faces appear in locations 3, 5, 7, 8, 12, 13.

We considered that each of these locations could hold a number of objects: i.e., faces of different ages at the higher locations (e.g. location 3, Fig. 6A), and a variety of furniture or clothes (objects) at the lower locations (e.g. location 6, Fig. 6A). The presence of an *antique*, a *modest* or a *common chair* at location 6 cues the *material circumstances* of the family: *wealthy*, *middle class* or *poor*. The presence of different faces cues the *average age* of the people in the picture: *young*, *middle aged* or *old*.

**Figure 7:**
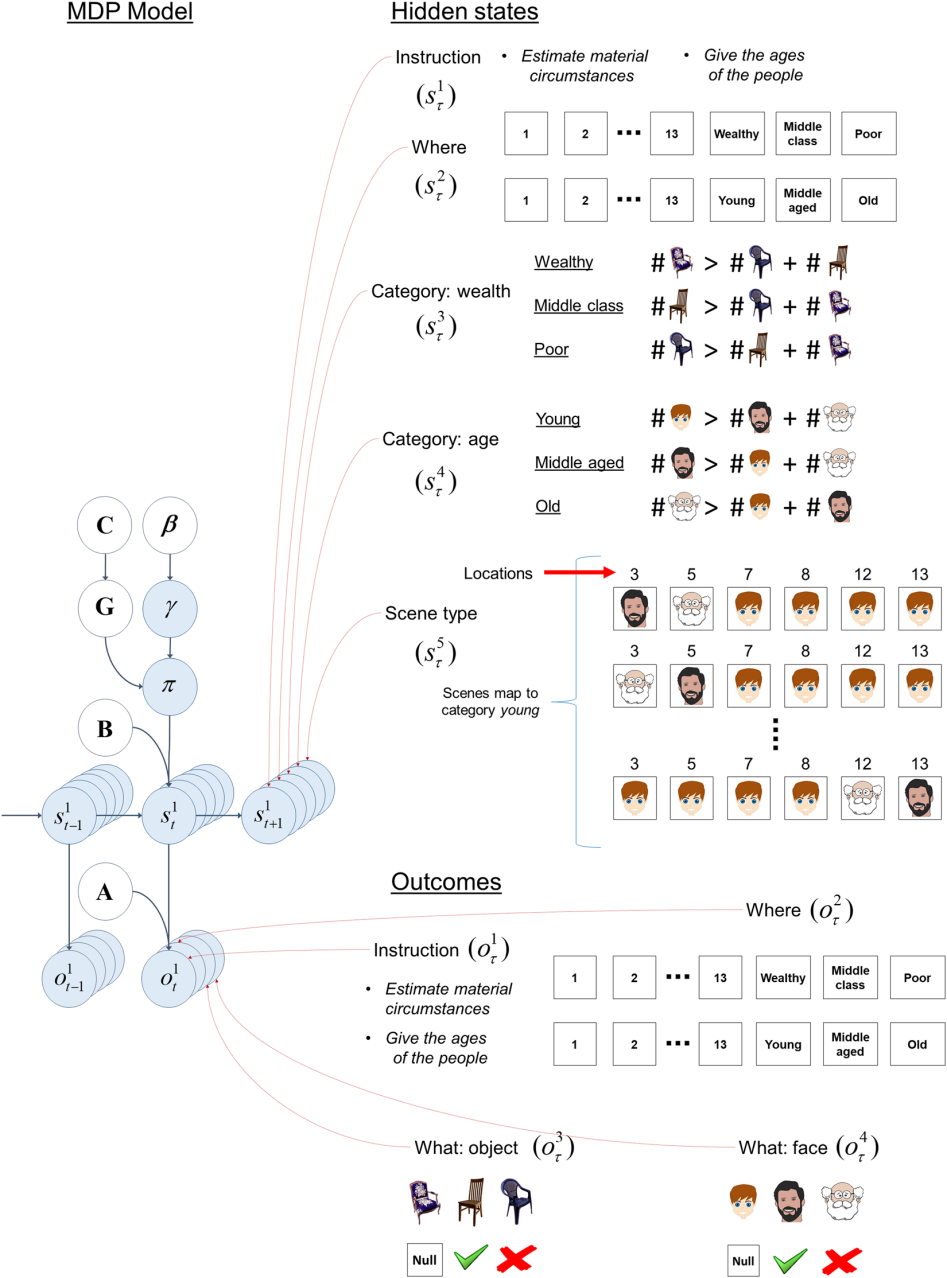
Structure of the generative model – Yarbus’ task. The structure of the generative model is shown on the left **–** see Fig. 3A for a detailed description. On the right are the five sets of hidden states and four outcome modalities in our MDP version of Yarbus’ task. There are five sets of hidden states, namely *Instruction*, *Where*, *Category: wealth*, *Category: age* and *Scene type*. There are four outcome modalities, namely *Instruction*, *Where*, *What: object* and *What: face*. See the main text for details. The cartoon faces and the pictures of the chairs in this figure have been downloaded from https://pixabay.com/.

The agent did not know the locations of the features in the scene. Initially, the agent held uniform beliefs over the features in each location and learned which feature is where by pursuing novel policies; see Eq. 4. These policies enable the agent to learn the (likelihood) mapping from locations in the scene to different features (objects and faces). Figure 6C shows how the agent learns the locations of the objects and faces (i.e., *What: object A*^3^ and *What: face A*^4^, see MDP model in the next section and Fig. 7) on an exemplar scene, described in Fig. 6B.

### MDP model

The hidden state and outcome spaces used in Yarbus’ task is formally similar to the Colour/Shape model described above. The hidden state space consists of five dimensions, namely *Instruction*, *Where*, *Category: wealth*, *Category: age*, *and Scene type*. *Instruction* is either *estimate material circumstances* or *give the ages of the people*. *Where* encodes one of thirteen locations in the scene. *Category: wealth* encodes the material circumstances, which could be *wealthy*, *middle class* and *poor*. *Category: age* encodes the average age of the people in the scene and these are *young*, *middle aged* or *old*. *Scene type* consists of a number of different scenes that map onto the same states in *Category: wealth* and *Category: age* state dimensions. Essentially *young*, *middle aged* and *old* faces can appear under different age categories. A scene whose category is *young* contains predominantly *young* faces. Each scene under the category *young* can contain other type of faces, e.g. *middle aged* and *old*. *Scene type* encodes the number and locations of these faces. *Scene type* encourages exploration of the scene. There are four outcome modalities, namely *Instruction*, *Where*, *What: object* and *What: face*. *Instruction* and *Where* states directly map onto *Instruction* and *Where* outcomes. *What: object* outcome contains *antique*, *modest* and *common* chairs, whereas *What: age* outcome contains *young*, *middle* aged and *old* faces. See Fig. 7 for the generative model.

### Simulations

In this setting, a very low precision would induce imprecise likelihood matrices for the task-irrelevant objects and categories: e.g., when the instruction is to estimate *material circumstances* of the family, the likelihood matrix for the *faces* becomes very imprecise and the likelihood matrix for the objects becomes very precise (*ζ*^*faces*^ = 0, *ζ*^*objects*^ → ∞).

**Figure 8:**
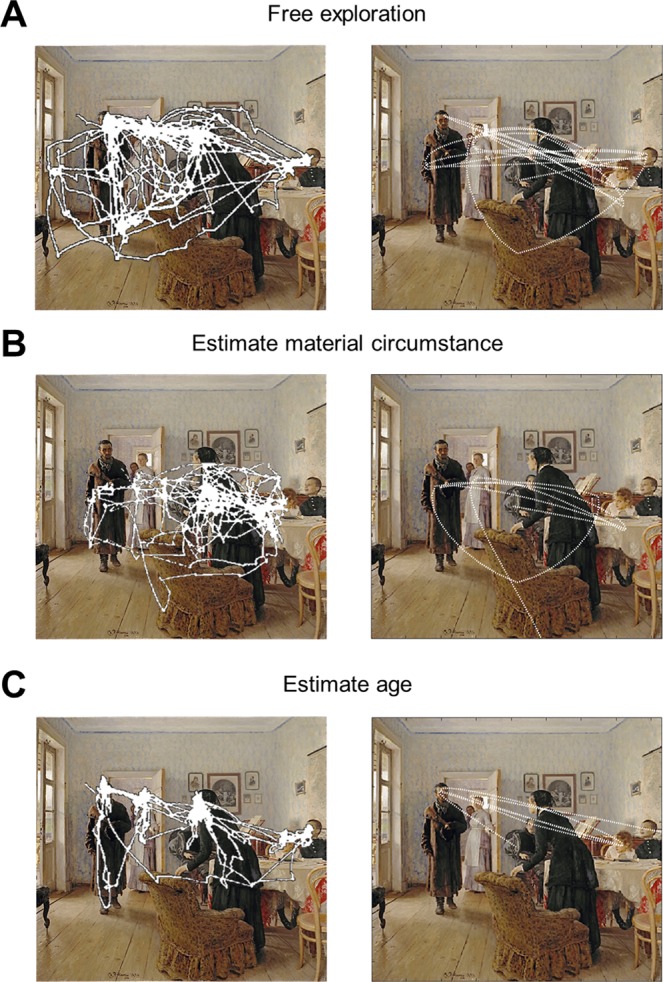
Yarbus’ free exploration task and simulations (**A**) The panel on the left shows how real subjects explored the painting freely in Yarbus’ experiment, whereas the right panel shows the simulated exploratory behaviour of a context naïve agent (unaware of the instructions and thus exploring the scene freely). The agent starts exploring from the centre of the scene (location 1) (**B**) The panels on the left and right show how the real subjects and the agent explore the painting under the instructions *estimate material circumstances* of the family. (**C**) The panels on the left and right show responses when exploring the painting under the instructions *give the ages of the people*. The painting Unexpected Visitors by Ilya Repin has been downloaded from https://commons.wikimedia.org/w/index.php?title=File:Ilya_Repin_Unexpected_visitors.jpg ^71^. The panels on the left show the scanpaths of the subjects that explored this painting in Yarbus’ work (see Yarbus’ work^6^ for the originals of the scanpaths). The scan-paths available on the below link are superimposed on the painting itself to produce the panels on the right: https://commons.wikimedia.org/w/index.php?title=File:Yarbus_The_Visitor.jpg ^72^.

The left panels of Fig. 8A–C show the saccadic scan paths recorded in Yarbus’ experiment, superimposed on the painting. The right panels show the simulated saccadic patterns generated using the principle above, illustrated for the colour/shape task. The agent was first allowed to explore the painting freely (Fig. 8A), then to estimate the material circumstances of the people (Fig. 8B), or their ages (Fig. 8C). Like Yarbus’ participants, the agent attends to all the faces and most of the furniture (and clothing) in the scene during free exploration (Fig. 8A); i.e., when the agent is unaware of the instructions, but when the instructions are *estimate the material circumstances of the family* or *give the ages of the people* the agent selectively attends to the furniture and clothing (Fig. 8B) or faces (Fig. 8C) respectively.

Here, we have offered a computational account that enables contextual modulation of visual exploration. There are a number of clinical conditions associated with atypical visual scanning, such as generalised anxiety disorder, schizophrenia and autism. These atypical exploratory behaviours have been illustrated in visual search paradigms, where top-down guidance of attention is crucial to perform visual tasks. In the section ‘simulating pathology’, we show that the relative precisions associated with context-relevant and irrelevant signals may account for the exploratory behaviours under anxiety and autism.

### Selective attention in Yarbus’ task

In Yarbus’ task, the precision of the sensory signals is modulated as a function of the *Instruction* hidden state dimension in the generative model. When the *Instruction* is *estimate material circumstances* the sensory precision of *What: face* outcomes becomes low, while the sensory precision of *What: object* outcomes becomes high, and vice versa for the hidden state *give the ages of the people* (or *estimate how long the unexpected visitor has been away* in the simulations of ASD, see the section ‘simulating pathology’). We can formulate a precision matrix similar to the matrix in Eq. 7,

8 $$\zeta =\begin{array}{c}{\boldsymbol{m}}\,\,\,\,\end{array}\begin{array}{c}Instruction\\ Where\\ What:object\\ What:face\end{array}\mathop{\mathop{[\begin{array}{cc}\,\,\,{\rm{\infty }} & \,\,\,\,\,\,\,\,\,\,\,\,\,{\rm{\infty }}\,\,\,\\ \,\,\,{\rm{\infty }} & \,\,\,\,\,\,\,\,\,\,\,\,\,{\rm{\infty }}\,\,\,\\ \,\,\,{\rm{\infty }} & \,\,\,\,\,\,\,\,\,\,\,\,\,z\,\,\,\\ \,\,\,z & \,\,\,\,\,\,\,\,\,\,\,\,\,{\rm{\infty }}\,\,\,\end{array}]}\limits^{\begin{array}{cc}Estimate & Estimate\\ wealth & age\\ \end{array}}}\limits^{{\boldsymbol{i}}}$$

When *z* = 0 the agent’s attention would be focused on only task-relevant visual stimuli, however when *z* → ∞ the agent’s attention would be focused on both task-relevant and irrelevant stimuli. For the simulated scanpaths (see the right panels of Fig. 8) under free exploration and exploration under instructions (estimate wealth or age), we chose *z* → ∞ and *z* = 0, respectively. For the simulated behavioural responses of typically developing (TD) and autism spectrum disorder (ASD), we chose *z* = 0 and *z* → ∞, respectively, see the section ‘simulating pathology’.

## Face Identification Task

In a visual task, people were asked to identify the emotion of a face presented on a computer screen as either happy or fearful. This task was performed under two conditions, namely *threat of shock* (threat or anxiety) and *free from threat of shock* (safe). Under the former, the participants anticipate a single electrical shock to their foot but are not told when this will be. The threat of shock induces anxiety. The two conditions instantiate *threat* and *safe* contexts. In the next section we will use this task to simulate the kind of behaviour observed in studies about anxiety.

### MDP model

**Figure 9:**
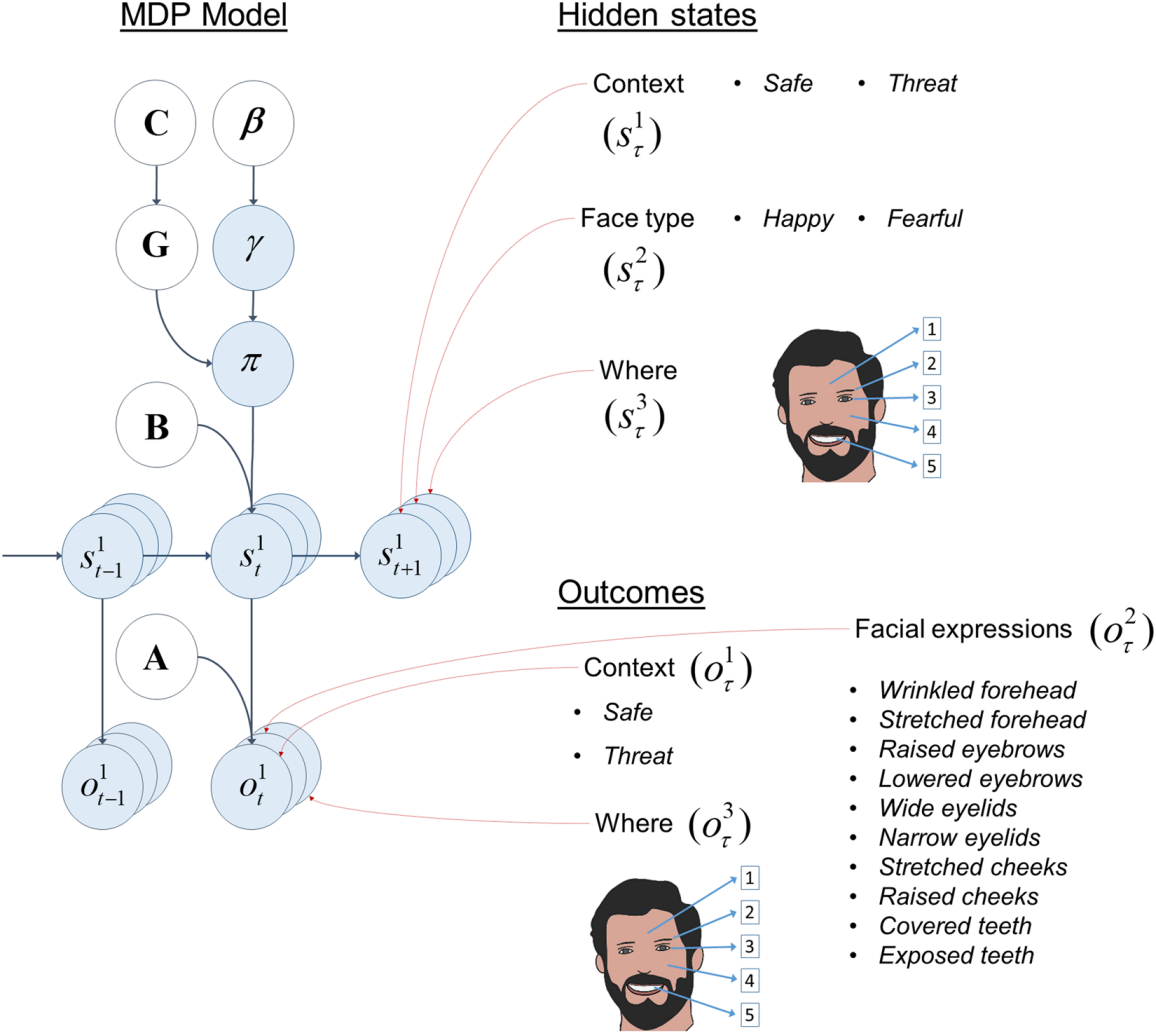
Structure of the generative model – Facial expression identification task. The structure of the generative model is shown on the left **–** see Fig. 3A for a detailed description. The right panel shows that there are three hidden states, namely *Context*, *Face type* and *Where*. There are three outcome modalities, namely *Context*, *Facial expressions* and *Where*. See the main text for details.

**Figure 10:**
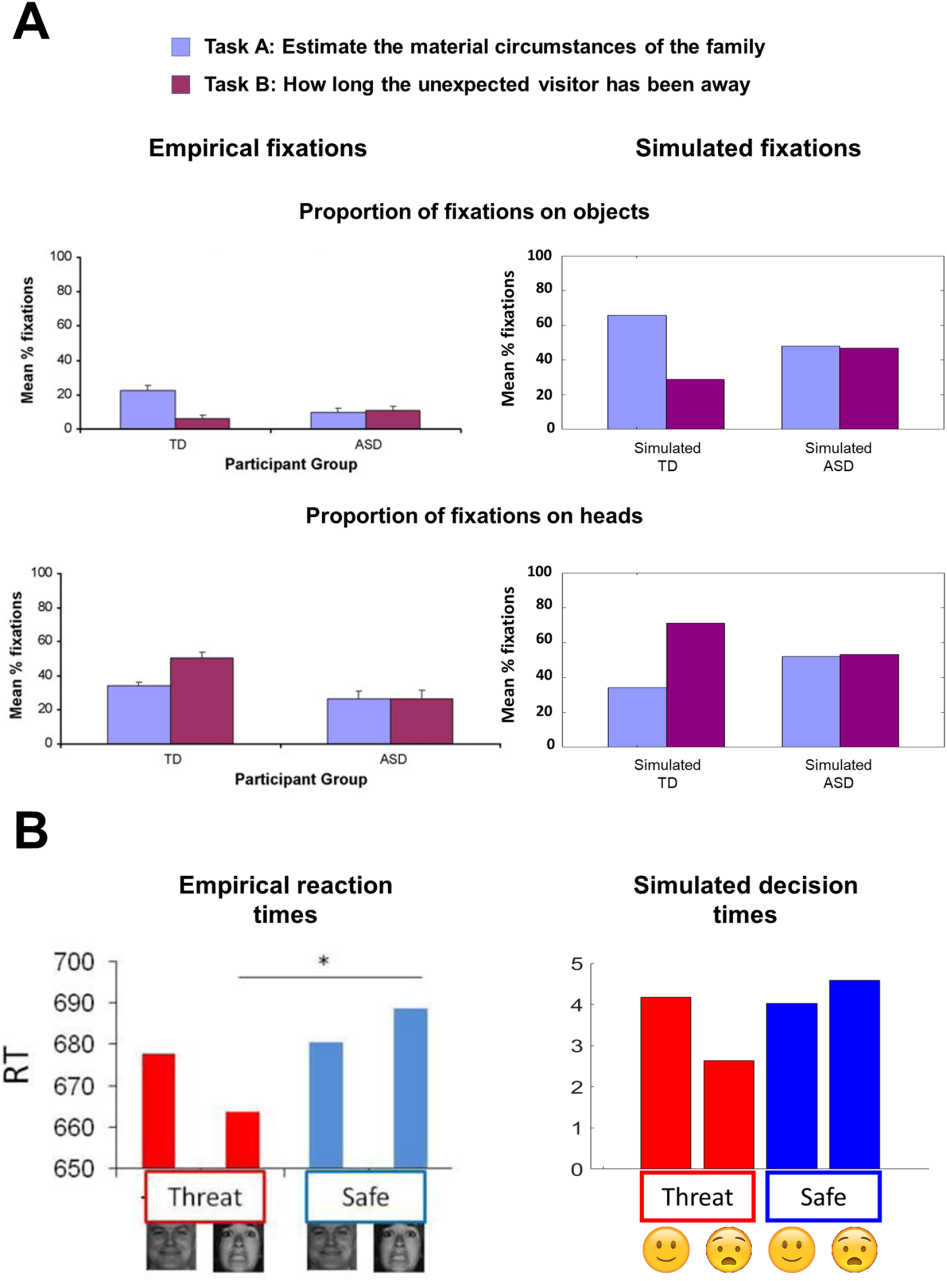
Empirical results and simulations (**A**) The left panels report the proportion of fixations on objects and heads when Yarbus’ visual search task is performed by typically developing (TD) adults and people with a diagnosis of autism spectrum disorder (ASD) under the instructions *estimate the material circumstances of the family* (Task A) and *estimate how long the unexpected visitor has been away* (Task B). The panels on the right show the simulated proportion of fixations using the MDP model. (**B)** Healthy people performed a face identification task under different conditions, namely *threat of shock* (threat) and *free from threat of shock* (safe). In this task, either a happy or a fearful face is shown on a computer screen. The participants are asked to identify the face as either happy or fearful as quickly as they can. The left panel shows the empirical reaction times (i.e., time it took to identify the facial emotion), whereas the right panel shows the simulated decision times using the MDP model (i.e., the number of facial features the agent attended before identifying the face). See the main text for details. See Benson *et al*.^35^ and Robinson *et al*.^37^ for the originals of the figures on the left panels of A and B in this figure, respectively. These figures were reproduced with permission from Elsevier and the rights to use these materials have been obtained through Copyright Clearance Centre.

The hidden state space consists of three dimensions, namely *Context*, *Face type* and *Where* (also see Fig. 9). Under the *Context* dimension the hidden states are *threat of shock* (threat or anxiety) or *free from threat of shock* (safe). *Face type* consists of two hidden states, namely *happy* or *fearful* face. Under the *Where* hidden state dimension there are five hidden states that correspond to different face areas, namely forehead, eyebrows, eyelids, cheeks and teeth. There are three outcome modalities, namely *Context*, *Facial expressions* and *Where*. The *Context* and *Where* hidden states map directly onto *Context* and *Where* outcomes, respectively. Happy and fearful faces are each associated with five facial attributes: fearful faces are associated with wide eyelids, a wrinkled forehead, etc, whereas happy faces are associated with exposed teeth, narrow eyelids, etc. The *Facial expressions* outcome modality is mediated by the *Face type* and *Where* hidden state dimensions (i.e. what type of face it is and where in the face one is looking). The full structure of the generative model used to simulate the results in the right panel of Fig. 10B is shown in Fig. 9.

### Enhanced perception in face identification task

For the simulated results about anxiety under ‘simulating pathology’ section, we used the following approach. In this task, the precision associated with facial expressions in fearful faces is modulated as a function of the *Context* hidden state dimension in the generative model. This works such that the precision associated with facial expressions in *fearful* faces increases in the *threat* context relative to the *safe* context, while leaving the precision of the facial expressions in the *happy* faces unchanged between contexts. We used the below precision matrices for the simulated responses in the right panel of Fig. 10B:

$${\zeta }_{f}={\boldsymbol{m}}\,\,\begin{array}{c}Context\\ Facial\,\,Expressions\\ Where\end{array}\mathop{\mathop{\mathop{[\begin{array}{cc}{\rm{\infty }} & {\rm{\infty }}\\ 0.1 & 0.9\\ {\rm{\infty }} & {\rm{\infty }}\end{array}]}\limits^{\begin{array}{cc}Safe & Threat\end{array}}}\limits^{{\boldsymbol{i}}}}\limits^{{\rm{F}}{\rm{a}}{\rm{c}}{\rm{e}}\,{\rm{t}}{\rm{y}}{\rm{p}}{\rm{e}}:\,fearful}{\zeta }_{h}={\boldsymbol{m}}\,\,\begin{array}{c}Context\\ Facial\,\,Expressions\\ Where\end{array}\mathop{\mathop{\mathop{[\begin{array}{cc}{\rm{\infty }} & {\rm{\infty }}\\ 0.25 & 0.25\\ {\rm{\infty }} & {\rm{\infty }}\end{array}]}\limits^{\begin{array}{cc}Safe & Threat\end{array}}}\limits^{{\boldsymbol{i}}}}\limits^{Face\,type:\,happy}$$

Here, Face type refers to the second hidden state dimension in the generative model (see Fig. 9). The precision matrices *ζ*_*f*_ and *ζ*_*h*_ show how the precision of the facial expressions change between safe and threat contexts in fearful and happy faces, respectively. The precision of facial expressions change from 0.1 (low precision) to 0.9 (high precision) from safe to threat context for the fearful faces. The precision of facial expressions in happy faces do not change between safe and threat contexts, and have a precision of 0.25 (medium precision).

Here, we described the precision manipulations used to simulate the behavioural responses of ASD and anxiety using Yarbus’ task and face identification task, respectively.

### Simulating pathology

#### Autism

Prominent theoretical accounts of autism spectrum disorder (ASD) call upon a failure to use prior beliefs to contextualise sensory data^30^. This is sometimes expressed as an enhancement of ‘bottom-up’ perception^31^ – i.e., elevated or unattenuated sensory precision^32^ – and sometimes in terms of weak (imprecise) priors that preclude top-down modulation of sensory signals^33^. Manifestations of these computational deficits include resistance to visual illusions, attenuated pupillary responses to *a priori* surprising stimuli^34^ and, crucially, abnormalities of visual search behaviour.

Here, we show that the exploratory saccadic behaviour of people with ASD is consistent with a failure to down-modulate (or attenuate) the precision of task-irrelevant sensory signals. This is motivated by an empirical study in which people with ASD and typically developing (TD) adults were asked to explore the same painting from Yarbus’ paradigm (Fig. 6A), under the instructions *estimate the family’s material circumstances* and *estimate how long the unexpected visitor has been away*^35^. Note that the only cues one can use to estimate how long the unexpected visitor has been away are the expressions on people’s faces in the painting (see the left panel of Fig. 6A).

In this task, the proportion of fixations on the objects were higher when the instruction was *estimate the family’s material circumstances* than when the instruction was *estimate how long the unexpected visitor has been away* for the TD adults (see the top left panel of Fig. 10A). Similarly, the proportion of fixations on the people’s heads were higher when the subjects were asked to *estimate how long the unexpected visitor has been away* than when the instruction was *estimate the family’s material circumstances* for the TD adults (see the bottom left panel of Fig. 10A). However, the proportion of fixations on objects and people’s faces did not change under these two instructions for the people with ASD (see the left panels of Fig. 10A). In our simulations, the responses of TD adults are reproduced by equipping the agent with the ability to attend away from task-irrelevant stimuli by attenuating the precision associated with these stimuli: e.g., the ability to attenuate the precision of objects when the instruction is *estimate how long the unexpected visitor has been away*. The simulated responses of the people with ASD were generated by an agent that is unable to attenuate the precision of task-irrelevant objects, which induces an inability to ignore task-irrelevant stimuli.

By modulating the capacity to attend away from irrelevant stimuli, we were able to simulate different patterns of visual search. The proportion of fixations on objects and faces during these searches are shown in the right panels of Fig. 10A). Although there are considerable differences between the simulated and empirical proportion of fixations on objects and faces (an artefact of our model only being able to fixate a small number of discrete locations in the scene), the main observation here is that TD and simulated TD adults are able to shift their attention to task-relevant objects, whereas people with ASD and simulated ASD are not. Although the second instruction used in the MDP model (*give the ages of the people*) differs from the second instruction in the empirical study (*estimate how long the unexpected visitor has been away*), both instructions require one to look at people’s faces in the painting and thus we made no changes in the MDP model to simulate these results (see Fig. 7 for more details).

#### Anxiety

Similar sorts of explanations have been leveraged to explain some of the perceptual features of states of anxiety. It has been suggested that there may be an enhanced sensory perception of anxiogenic stimuli (e.g., pictures of fearful faces) in anxiety. The evidence for this comes from studies employing ‘threat of shock’ conditions^36,37^.

In these studies, people identify fearful faces faster in the *threat* context than in the *safe* context (see Fig. 10B left panel). This suggests that the facial expressions in a fearful face may be perceived as delivering more information about the emotion of the face under the threat of shock than when there is no threat. This would explain why fearful faces are identified quicker under the threat context.

We can use the same approach used to simulate the saccadic patterns on the Yarbus task to produce similar behavioural responses – as illustrated in the left panel of Fig. 10B. The visual stimuli in this case could either be a fearful or a happy face and the agent’s task is to find out what type of face it is looking at. In this task, the context could either be *threat* or *safe*. Having an enhanced perception of anxiogenic stimuli translates to the ability to increase the precision associated with the facial expressions in fearful faces (wide eyelids, raised eyebrows etc.) under the *threat* context relative to the *safe* context in the MDP model. This means that the agent would need to observe only a few facial components before identifying the face as a fearful face. The empirical reaction times are approximated in simulations with the decision times, which are the number of locations looked at before identifying the face. The key observations from the simulations are: (i) the fearful faces are identified faster in the *threat* context than the fearful faces in the *safe* context, and (ii) identification of happy faces was the same under both *threat* and *safe* contexts (see the right panel of Fig. 10B). This is because only the precision of the fearful-*threat* mapping is adjusted between contexts (see Fig. 9 and the face identification task in the previous section). One might query why the precision associated with the facial expressions in *happy* faces and safe contexts does not appear to be modulated between contexts. It seems plausible that a threat context induces two effects: (i) an increase of the precision associated with the facial expressions in fearful faces and a decrease of the precision associated with the facial expressions in happy faces, and (ii) a generalised increase in sensory precision of all such mappings. This would produce the pattern we see in Fig. 10B.

## Discussion

Selective attention is often divided into two categories: overt attention (i.e. performing a motor act to orient to a stimulus), and covert attention (where no action is performed). Our focus has been on the role of context in influencing overt saccadic behaviour. However, there is an important covert element to this. The process of ascribing more or less precision to different locations does not require a movement and could be thought of as the deployment of covert attention. In this sense, the behaviour illustrated in this paper may be thought of as showcasing how covert attention drives overt attentional sampling.

In this work, we have provided proof of principle that an agent can selectively attend to information that is useful – under a particular context – by inferring the appropriate attentional targets. Computationally, this corresponds to modulating the precision of the mapping (encoded by the likelihood matrix) between task-irrelevant sensory inputs (stimuli that are not informative in a certain context) and their hidden causes. When the precision of the task-irrelevant likelihood is low, an agent only attends to task-relevant stimuli. This model reproduces the saccadic patterns in empirical studies of context-dependent human exploratory behaviour^6^.

The exploratory behaviour of the agent described in this work is driven by epistemic value^4^, a.k.a. (expected) Bayesian surprise. Bayesian surprise attracts human attention^15^: in other words, a stimulus attracts attention if it changes an observer’s beliefs significantly. Clearly, this depends upon what beliefs an observer currently holds. We have demonstrated a capacity to revaluate beliefs about context, given a cue, such that the same stimulus can carry different levels of surprise in different contexts.

Most computational models of visual search are bottom-up models of visual attention that do not consider the contextual information inherent in visual scenes. These models usually create a ‘saliency map’ based on the features of the objects in the scene. These features include orientation, intensity, colour information^38–41^, luminance^42^, contrast^43^ and motion^44^. Typically, the locations in these saliency maps are attended in order of decreasing salience – often requiring an inhibition-of-return rule to make simulations work plausibly. Although these models provide relatively good predictions of where visual attention will be deployed in pop-out visual search tasks, they do not incorporate contextual information. There is no reason why a bottom-up visual search model would find the faces more salient when an instruction such as ‘give the ages of the people in the scene’ is given. Only models with a top-down aspect have the potential to make use of such instructions.

There are a number of visual attention models that can incorporate top-down knowledge during visual search. Top-down instructions in these models are usually given in the form of prior knowledge about the features of an object of interest. While some top-down models evaluate the similarity (or dissimilarity) of the features of the object of interest, with the features in the scene that is being explored^45^, there are other models that either modulate or select feature outputs – such that the features of the object of interest become more salient^46,47^. A noteworthy model in this setting defines image categories in terms of visual patterns and approaches the scene categorisation problem by maximising the mutual information between scene categories and pixel values at possible fixation locations^48^. A similar approach maximises the pointwise mutual information between a target object and visual features^49^. There are also other top-down models that either use iconic scene representations – to predict the location that holds the object of interest^50^ – or models that equate salience to discrimination and consider the features that best distinguish the object of interest from the other objects as salient^51^.

The model we have presented illustrates a computational link between attentional control and the efficient sampling of information. As with some of the models above, our model is equipped with an information acquiring imperative; namely, epistemic value. Epistemic value resolves uncertainty about the hidden states of the world and corresponds to the mutual information between hidden states and observations. Our model distinguishes itself from the above formulations in a number of ways. In our formulation of selective attention, task-relevant exploration arises by entertaining imprecise beliefs about context-irrelevant objects. This precludes information gain about task-irrelevant hidden states (e.g., scene categories). To our knowledge, this is the first attempt to model selective attention in terms of context-sensitive epistemic affordance. Furthermore, our model can successfully report its beliefs about the scene category by exploiting extrinsic value (e.g., expected utility). Finally, our formulation emphasises top-down inferential processes that use relatively abstract (e.g., semantic) representations. This contrasts with the lower level representations used in other models to describe features of visual scenes. In other words, the epistemic value is an expected Bayesian surprise that pertains to beliefs about hidden states of the world – as opposed to visual features.

While we have not addressed the contributions of early visual pathways here, we could interpret our sensory outcomes as alternative hypotheses about continuous attributes of visual objects^52^. Active inference calls on an explicit generative model that depends upon prior beliefs. This is important, as a number of clinical conditions have been associated with aberrant prior beliefs, and this paradigm might afford an opportunity to investigate these conditions quantitatively. People with a diagnosis of autism spectrum disorder (ASD) are known to explore visual scenes (especially faces) differently than neurotypicals. In free visual search tasks – that contain pictures of faces – people with ASD attend less to the core features of faces (e.g., eye, nose and mouth) and more to other parts of the face^53^ and are slower at discriminating faces in face discrimination tasks^54^. In contrast, people with ASD have been shown to be superior to controls on visual search tasks that involve visual illusions^55^ and faster on tasks that involve spotting a target object that shares certain features with distractors^56^.

The visual foraging of people with ASD may be due to one or more perturbations under our model: altered model structure (e.g., not knowing the mapping from gaze to mental states), reduced recognition of context (e.g., not realising that a given situation warrants information gathering about mental states); where context can be defined as the global configuration of features and objects, or a difficulty in down-modulating the precision of task-irrelevant object mappings. Our simulations suggest that the last perturbation could account for the epistemic behaviour of people with ASD, on free viewing visual search tasks under different instructions (although carefully designed experiments are required to disambiguate the three perturbations above). Interestingly, a difficulty in down-modulating precision would also imply a more accurate generative model – consistent with superior (pop-out) visual search performance in autism^57^.

Autism is not the only condition that has been associated with abnormal precision weighting. The aberrant salience hypothesis of schizophrenia proposes that altered attribution of salience to sensory stimuli may underwrite perceptual and attentional changes in psychosis^58^. Aberrant attribution of salience may be exacerbated by deficits in context processing^59^, but there is thought to be a predominant impairment in the control of attention (i.e., feature selection) in schizophrenia than in the subsequent inference using those features^60^. Indeed, whilst subjects with schizophrenia may be unimpaired – or even show enhanced performance – in simple attentional cueing tasks^61^, in more complex tasks, such as viewing natural images, they consistently fixate less on informative areas^62^. Thus, it may be that problems with context recognition and control of precision modulation contribute most to schizophrenia, whereas in ASD a lack of key model structure (about the mental states of others) may be more important.

In studies where anxiety is induced by threat of shock, people identify fearful faces faster when anxious. It has been suggested that anxiety could have adaptive value in dangerous situations^63^. Enhanced sensory perception of stimuli that predict imminent danger could certainly be adaptive, as this would allow one to react quickly to dangerous situations. We can interpret this in terms of a (possibly evolutionary derived) prior belief about how to contextualise visual perception in relation to such situations. Here, we have shown that the *threat* context modulates the precision of threat-related stimuli (fearful faces), which leads to faster identification of these stimuli.

Our focus in this paper was on providing a proof of principle that selective attention can be modelled in terms of active inference – in a way that highlights its close relationship to constructs in psychology, psychophysics and computational neuroscience. We did not compare the performance of this model to normative (i.e., descriptive) models of related phenomena because in this work we were concerned with understanding selective attention from first principles. Because these principles encompass Bayes optimality, the performance of the model described in this paper is, by definition, optimal. Clearly, this does not address the issue of whether this is a good model of human behaviour (or electrophysiological responses). Although we addressed this issue anecdotally by appealing to classical results in the literature, this paper restricted itself to simulations and proof of concept. In subsequent papers we will fit the model to empirical (e.g., eye tracking) data. The question in this setting reduces to what priors does any particular subject bring to the table – that best explains their responses – under the generative model described in this work.

Here we considered the problem of using contextual information to drive a form of selective attention where we have knowledge of the scene under question. The prior knowledge about the scene is acquired through initial scan of the scene as shown in Fig. 6C.

The model that we describe here uses purely foveal visual outcomes rather than incorporating a larger receptive field. While this was sufficient to address the sort of context-driven selective attention we sought to understand, it is clearly an oversimplification. The limitations of this choice are exposed if we consider the kind of attentional processes required in a visual search paradigm that involves locating specific stimuli in an array, with no prior exposure to this array. In this setting, people are able to locate the target in a few eye movements^10,64–66^, and show electrophysiological responses to these stimuli within hundreds of milliseconds^67^. Clearly this is a much faster time-course than that of the initial exposure to (and learning of) the stimulus that we used and implies the use of peripheral retinal input to draw inferences about where to look.

For an example of how multiscale and multiresolution sensory input may be incorporated into a generative model of the sort used here, please see^27,68^. These approaches include high resolution foveal and low-resolution peripheral vision.

To model visual search tasks that mandate finding a stimulus in an array would require a generative model with ‘location’ hidden states for each object, and that predicts both central and peripheral visual outcomes based upon the fixation location and object locations. This could in principle accommodate ‘straight to the target’ behaviour in visual search tasks without the need for learning. An alternative would be to infer only the second order statistics associated with different peripheral spatial locations, without having to make explicit predictions about the content^69^. This sort of inference, thought to underwrite phenomena like figure-ground segregation, implicitly estimates the ambiguity of each location in space, an important component of salience. This has the potential to drive saccades to locations in peripheral vision through an apparently exogenous attentional process.

This work has some limitations. The visual search tasks that we have considered are fairly simple tasks. More complicated tasks – that involve viewing of natural scenes – may have many more rules (or instructions) to consider than the contingencies considered in our tasks. We have not attempted to distinguish the potential causes of aberrant exploratory behaviours in ASD and schizophrenia. Nevertheless, this MDP model of active inference has the potential to differentiate between abnormal behaviours with distinct causes^70^.

## Conclusion

This theoretical work has illustrated the computational mechanisms that may underwrite selective attention that contextualises visual exploration and expected information gain. Contextual exploration requires attentional mechanisms that highlight relevant sources of information. Under active inference, attention can be thought of as the precision of sensory signals given their hidden causes. We appealed to this aspect of active inference by making the precision of the likelihood mapping between sensory signals and their hidden causes context-dependent. This allowed us to show that context-driven exploration arises as a result of down-weighting the precision of the context-irrelevant sensory signals, while maintaining the precision of the context-relevant sensory signals.

## Data Availability

The simulation results shown in this paper were produced using a standard software routine, **s**pm_MDP_VB_X.m. This matlab code is available in the SPM software: http://www.fil.ion.ucl.ac.uk/spm/
